# Biomarker Discovery for Early Detection of Pancreatic Ductal Adenocarcinoma (PDAC) Using Multiplex Proteomics Technology

**DOI:** 10.1021/acs.jproteome.4c00752

**Published:** 2024-12-19

**Authors:** Alcibiade Athanasiou, Natasha Kureshi, Anja Wittig, Maria Sterner, Ramy Huber, Norma A. Palma, Thomas King, Ralph Schiess

**Affiliations:** † Proteomedix AG, Wagistrasse 23, CH-8952 Schlieren, Switzerland; ‡ Immunovia Inc., 26 Forest Street, Suite 110, Marlborough, Massachusetts 01752, United States; § Immunovia AB, Medicon Village, Scheelevägen 8, SE-223 63 Lund, Sweden

**Keywords:** pancreatic cancer, PDAC, Olink, protein biomarker, quantitative proteomics

## Abstract

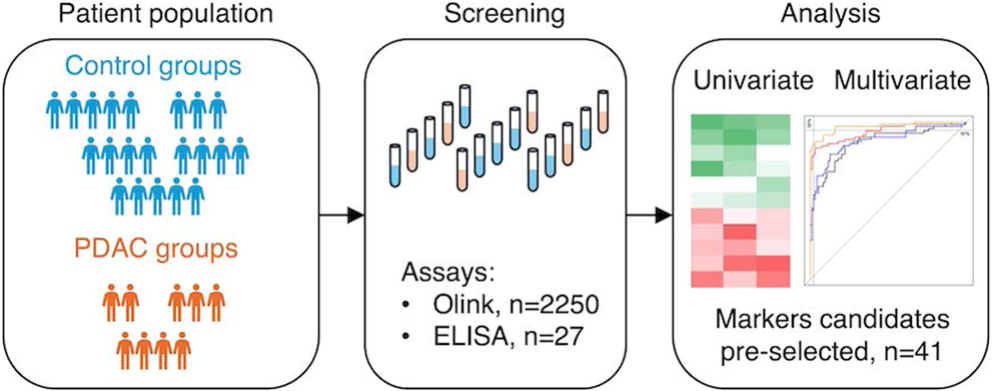

Early detection of pancreatic ductal adenocarcinoma (PDAC) can improve survival but is hampered by the absence of early disease symptoms. Imaging remains key for surveillance but is cumbersome and may lack sensitivity to detect small tumors. CA19-9, the only FDA-approved blood biomarker for PDAC, is insufficiently sensitive and specific to be recommended for surveillance. We aimed to discover a blood-based protein signature to improve PDAC detection in our main target population consisting of stage I or II PDAC patients (*n* = 75) and various controls including healthy controls (*n* = 50), individuals at high risk (genetic and familial) for PDAC (*n* = 47), or those under surveillance for an intraductal papillary mucinous neoplasm (*n* = 36). Roughly 3000 proteins were measured using Olink multiplex technology and conventional immunoassays. Machine learning combined biomarker candidates into 4- to 6-plex signatures. These signatures significantly (*p* < 0.001) outperformed CA19-9 with 84% sensitivity at 95% specificity, compared to CA19-9’s sensitivity of 53% in the target population. Exploratory analysis was performed in new-onset diabetes (*n* = 81) and chronic pancreatitis (*n* = 50) patients. In conclusion, 41 promising biomarker candidates across multiple signatures were identified using proteomics technology and will be further tested in an independent cohort.

## Introduction

Over 60,000 people in the United States will be diagnosed with pancreatic cancer in 2024.^1,2^ In the vast majority of patients, their condition is detected too late to be treatable by surgery, resulting in a 5-year survival rate of less than 13%.^3,4^ Earlier detection of pancreatic cancer can improve survival, but unfortunately, pancreatic ductal adenocarcinoma (PDAC), the most common form of pancreatic cancer, usually shows few or no symptoms in its early stages.^5,6^ The National Comprehensive Cancer Network (NCCN), the American Gastroenterological Association, and the International Cancer of the Pancreas Screening Consortium support surveillance for individuals at high risk for pancreatic cancer because of their family history and/or predisposing pathogenic germline variants.^7^ Currently, surveillance is accomplished through diagnostic imaging (magnetic resonance imaging, MRI, or endoscopic ultrasound, EUS), but these procedures are potentially invasive and are relatively insensitive for the detection of small tumors.^8−10^ The availability of a sensitive, blood-based biomarker test for early PDAC detection could greatly improve this clinical situation and positively impact patient survival.^11,12^

The carbohydrate CA19-9 is the only clinically available biomarker that is used to monitor therapy/recurrence in PDAC patients, but it is not sufficiently sensitive or specific to be recommended for the surveillance of high-risk individuals.^13−15^ CA19-9 expression also varies in different ethnic populations further limiting its applicability for surveillance.^16−18^ No other single biomarker has emerged with sensitivity and specificity superior to CA19-9.^19,20^ Therefore, the identification of new biomarkers detectable in early-stage PDAC is needed to develop a blood test with sufficient sensitivity and specificity for clinical use.^21^

We set out to identify new biomarkers that in combination could potentially fill this clinical need. The aim of this study was to identify promising biomarker candidates associated with stage I or II PDAC. Roughly 3000 proteins were analyzed using the Olink Proximity Extension Explore Assay (PEA) platform^22^ in combination with targeted enzyme-linked immunosorbent assay (ELISA) for proteins reported to be associated with PDAC. This approach allowed us to identify 41 potential candidates, assess the correlation between Olink and ELISA results, and assess the level of correlation of expression between identified candidates in preparation for the development of a multianalyte signature for the early detection of PDAC.

## Materials and Methods

### Patient Population

The retrospective cohort included 352 samples selected from 6 countries (Denmark, Spain, Finland, Germany, Sweden, and USA) across 13 different sites (Table S1). All of the data were fully anonymized before they were accessed. The study was approved by the local ethics committees, and all patients gave written informed consent. Patients were divided into two main groups: controls and cases. For the cases group, all patients were clinically or pathologically staged stage I or II PDAC. Cases were either familial, genetic, sporadic, or patients with new-onset diabetes (NOD) and confirmed treatment-naïve PDAC. For the controls group, the following patients were selected: healthy controls (HC), patients with chronic pancreatitis (CP), those with intraductal papillary mucinous neoplasm (IPMN), patients with new-onset diabetes (NOD), and finally high-risk individuals (HRI), the latter being patients with a family history of PDAC or genetic predispositions.

### Olink and Targeted Immunoassay Method

Clinical samples were sent to TATAA Biocenter, Göteborg, Sweden for Olink PEA measurements using the “Explore 3072” panel covering 2926 proteins. The Olink data are presented as normalized protein expression (NPX) values. NPX is Olink’s relative protein quantification unit on log2 scale and is calculated from the number of matched counts, using NGS (Next Generation Sequencing) as readout. The following parameters were evaluated in the Quality Control (QC): (1) the average matched counts for each sample, where there should be at least 500 counts; otherwise, the sample receives a QC warning status. (2) The deviation of the median value of the negative controls from a predefined value set for each assay. To pass QC, the deviation of the median of the Negative Controls must be less than or equal to 5 standard deviations from the set predefined value; otherwise, the assay will receive a warning status.

Of the 2926 Olink proteins measured, there were 1687 (58%) proteins with no missing NPX values. In addition, 568 (19%) proteins had ≤20% missing NPX and were imputed using random forest^23^ and included for analysis. Finally, 5 proteins did not pass QC and were removed from the analysis panel. Thus, measurement of serum samples by Olink resulted in 2250 (77%) proteins (list of proteins in Table S2) used in the final statistical analysis. Principal component analysis (PCA) plots of samples by site were used to investigate outliers among the samples, especially those flagged by the Olink. None of the samples flagged by Olink were identified as outliers in the PCA plots and were left for analysis. However, one sample, an NOD patient, was identified as an outlier and was removed from analysis. Thus, the final population with a complete data set used for further analysis consisted of 329 patients.

In addition to Olink data, published data was reviewed to identify proteins reported to be associated with pancreatic ductal adenocarcinoma (PDAC). From this list, 27 proteins reportedly associated with PDAC were selected. 330 of the 352 samples were measured with this set of 27 markers using targeted immunoassays, as listed in Table S3. Commercially available assays include ACRP30, CA50, CA125, CA153, CA19-9, CEA, DLK1, ECM1, GAL3BP, IL1Ra, IL6, LAMC2, LCN2, LRG1, LUM, MMP9, NCAM1, OPG, OPN, THBS2, TIMP1, TTR, and ZAG. The other markers were based on proprietary antibodies from Proteomedix, specifically CTSD, ICAM1, POSTN, and THBS1. The calibration of the targeted immunoassays is based on proprietary recombinant proteins from Proteomedix for CTSD, ICAM1, and THBS1, while commercially available recombinant proteins were used for all other markers. For the measurement protocol, serum samples were measured in technical duplicates to ensure accuracy, and two control sera were used as longitudinal controls to track the variations between the assay runs. Measurements with an intra-CV > 15% were repeated.

In summary, for the immunoassay measurements, inter-CVs of two longitudinal controls were used to assess the run-to-run variation of the assay. Six of the 27 assays showed high inter-CVs (>15%, CA50, DLK1, IL1Ra, OPG, POSTN, and THBS2), while two had an acceptable CV (10–15%, ACRP30 and ZAG) and all other 19 assays had good inter-CVs (<10%).

### CA19-9 and IMMray PanCan-d method

The measurement of IMMray PanCan-d, a multiplex immunoassay that combines measurements of 9 serum biomarkers including proteins measured by eight single-chain variable fragment antibodies and CA19-9 using a mathematical algorithm,^10^ was performed in the CLIA-certified laboratory of Immunovia, Inc., in Marlborough, MA. Serum samples were analyzed with an antibody microarray platform composed of 8 single-chain variable fragments directed against 8 antigens after biotinylation (NHS-PEG4-Biotin No-Weigh Format; Thermo Fisher Scientific, Waltham, MA). The biotinylation reagent was quenched with Tris HCL, pH 7.5, and samples were diluted in phosphate-buffered saline containing Tween and dissolved milk powder as a blocking agent before being pipetted onto microarrays. Each sample was analyzed in duplicate on blocked arrays on resin-coated slides (Thermo Scientific Nunc; Immunovia AB). After incubation, arrays were individually washed and then incubated with the Streptavidin-Alexa Fluor 647 conjugate (Molecular Probes). After washing, the array slides were dried and immediately scanned using an InnoScan 710 AL (Innopsys) microarray scanner. Measurement of CA19-9 was performed with Luminex (Merck Miliplex kit, HCCBP1MAG-58K) according to the manufacturer’s instructions.

### Statistical Methods

The discriminative ability of markers (univariate) between controls and PDAC was evaluated by using a two-sided *t* test. Two approaches were employed to train the combinations, independently of the univariate performance: (1) all markers were used for training numerous “Generalized Linear Model” (GLM) multivariate combinations, and (2) only markers measured using immunoassay were used. Combinations were trained using various patient controls versus case groups. Performance assessment was carried out in the whole cohort and in different patient subpopulations, using sensitivity at 95% specificity as the key parameter.

All possible combinations of four to six parameters were used to train Generalized Linear Models (GLM) with PDAC as the binary outcome. Since the number of parameters in the models (4–6) was relatively large compared to the number of PDAC cases (22–75 depending on the population), Elastic Net regularization was applied to handle high multicollinearity and improve the model’s prediction ability. Additionally, combinations were developed, including only immunoassay-detected markers. These different combination algorithms were trained using the entire population as well as different subpopulations. The performance of the combinations was then assessed by applying the algorithms to the subpopulations and evaluating their sensitivity at 95% specificity.

When comparing sensitivities and specificities for paired tests, differences were evaluated using the McNemar test.^24^ AUC from ROC were compared using the Delong method.^25^ For all tests, *p*-values below 0.05 were deemed statistically significant. All statistical analyses were performed using R statistical packages, version 4.0.2.

## Results

### Population

Demographics of the 329 patients across the groups are listed in Table 1: PDAC cases were categorized as NOD (*n* = 22), PDAC stage I (*n* = 35), or PDAC stage II (*n* = 47), resulting in a total of 97 cases. Out of the 97 PDAC cases, 31 patients showed low CA19-9 (<37 U/mL) values. Controls were divided into CP (*n* = 50), HC (*n* = 50), HRI (*n* = 47), IPMN (*n* = 36), and NOD controls (*n* = 49), providing 232 controls in total. The percentage of females and males in the cohort was nearly evenly distributed, at 49.5 and 50.5%, respectively, and the average patient age was 64. In this study, performance assessment was conducted across the whole population, a “target” population, CP population, and an NOD population. The target group included HRI, HC, and IPMN as controls, and patients with stage I or II PDAC as cases. The CP group included only CP controls and stage I or II PDAC cases. The NOD group consisted only of NOD patients, diagnosed either with or without PDAC. Finally, the low CA19-9 subpopulation comprised samples from 128 patients with low CA19-9 (<37 U/mL).

**Table 1:** Demographic of the Patients across All Groups^a^

group	patients, *n* (%)	subpopulation	age, mean (SD), range	CA19-9, mean (SD), range	female, *n* (%)
all data	329 (100%)		64 (11), 25–86	73 (246), 0–2175	163 (49.5%)
controls CP	50 (15%)	CP	60 (9), 46–85	35 (67), 2–472	20 (40%)
controls HC	50 (15%)	target	57 (7), 40–70	9 (9), 0–53	21 (42%)
controls HRI	47 (14%)	target	54 (11), 25–75	17 (22), 1–113	25 (53%)
controls IPMN	36 (11%)	target	67 (8), 47–79	22 (20), 1–74	21 (59%)
controls NOD	49 (15%)	NOD	68 (10), 50–86	60 (309), 1–2175	28 (57%)
PDAC NOD	22 (7%)	NOD	72 (7), 52–85	188 (291), 4–975	9 (41%)
PDAC stage I	36 (11%)	CP, target	69 (7), 57–83	79 (94), 0–371	20 (56%)
PDAC stage II	39 (12%)	CP, target	70 (12), 33–86	259 (532), 4–2175	19 (49%)

a CP = Chronic pancreatitis, HC = Healthy control, HRI = High-risk individual, IPMN = Intraductal papillary mucinous neoplasm, NOD = New-onset diabetes, PDAC = Pancreatic ductal adenocarcinoma, Target = HRI, HC, IPMN controls and stage I or II PDAC cases.

### Olink and Immunoassay Analysis

**Figure 1:**
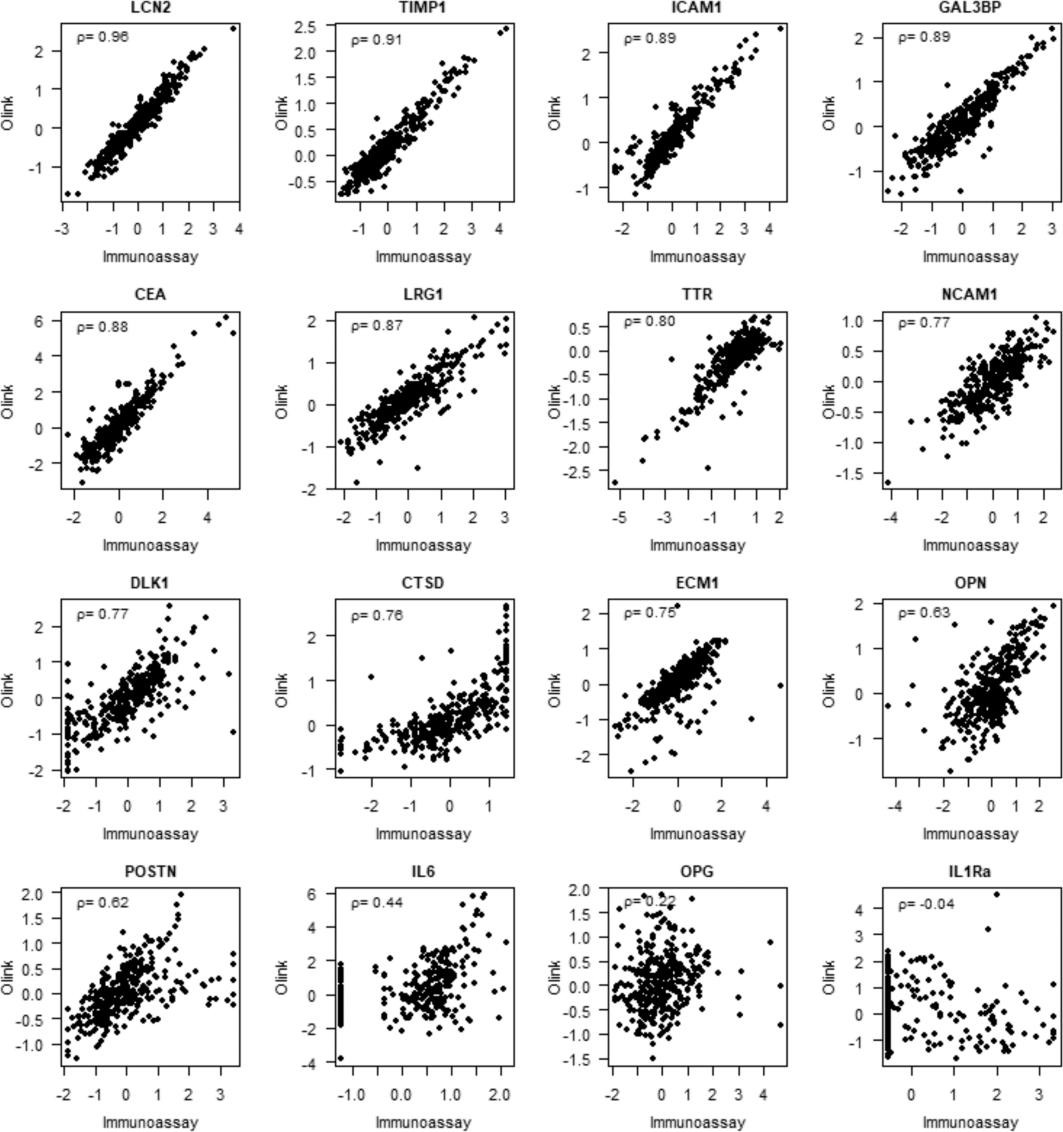
Correlation plots of protein analyzed by Olink and immunoassay. Spearman’s rho *p*-value is presented in the top left corner of the plots.

Quantitative proteomics analysis by Olink and targeted immunoassay analysis resulted in 2261 individual proteins quantified in serum. For 16 proteins, both the Olink and immunoassay data were available. Based on Spearman correlation coefficient, 8 proteins showed good correlation including GAL3BP (0.89), ICAM1 (0.89), LCN2 (0.96), LRG1 (0.89), TIMP1 (0.91), TTR (0.80), and CEA (0.88), another 5 showed medium correlation including ECM1 (0.75), CTSD (0.76), DLK1 (0.77), NCAM1 (0.77), POSTN (0.62), and OPN (0.63), while IL1Ra (−0.04), OPG (0.22), and IL6 (0.44) showed no correlation (Figure 1).

### Univariate Analysis

Univariate analysis was performed to differentiate PDAC cases from controls by examining sensitivity at fixed 95% specificity and *p*-values in the target (*n* = 208), CP (*n* = 125), and NOD (*n* = 71) populations. The results can be found in Table 2. In the target population, a total of 41 markers showed statistically significant sensitivity in one of the subpopulations. Among these, in the target population, nearly all markers had a lower sensitivity compared to that of CA19-9 (53%). CTSD (53%) and ICAM1 (52%) matched CA19-9′s sensitivity, while other markers such as MERTK (48%), TIMP1 (45%), PLA2G1B (44%), GPNMB (44%), and TR10A (44%) showed slightly lower sensitivity at equal specificity of 95%.

**Table 2:** Univariate Analysis of the Markers in the Different Subpopulations^a^^,^^b^

			sensitivity (%)			*p*-value		
protein	Olink gene	assay format	target at 95% spe.	CP at 96% spe.	NOD at 96% spe.	target	CP	NOD
CA19-9		IA	53	31	41	<0.001	0.001	0.001
CTSD		IA	53	12	18	<0.001	0.421	0.458
ICAM1		IA	52	16	14	<0.001	0.149	0.351
MERTK	Q12866	Olink	48	32	23	<0.001	<0.001	0.044
TIMP1	P01033	Olink/IA	45	4	5	<0.001	0.718	0.938
GPNMB	Q14956	Olink	44	27	14	<0.001	0.002	0.081
PLA2G1B	P04054	Olink	44	12	18	<0.001	0.033	0.209
TR10A	O00220	Olink	44	5	5	<0.001	0.844	0.725
MUC13	Q9H3R2	Olink	43	36	14	<0.001	0.001	0.268
ANGTPL2	Q9UKU9	Olink	43	1	9	<0.001	0.380	0.237
TTR		IA	41	3	14	<0.001	0.783	0.500
SEMA7A	O75326	Olink	40	27	14	<0.001	0.060	0.551
CLPS	P04118	Olink	40	9	18	<0.001	0.075	0.270
LTBP2	Q14767	Olink	39	0	9	<0.001	0.591	0.185
CEA		IA	36	13	18	<0.001	0.584	0.108
MMP7	P09237	Olink	36	7	14	<0.001	0.069	0.409
CBPB1	P15086	Olink	35	7	23	0.008	0.392	0.040
PTPRK	Q15262	Olink	33	16	5	<0.001	0.408	0.839
FGL1	Q08830	Olink	33	7	9	<0.001	0.057	0.347
GPA33	Q99795	Olink	32	7	14	<0.001	0.397	0.244
CTRL	P40313	Olink	31	8	18	0.947	0.575	0.067
PI16	Q6UXB8	Olink	29	7	5	<0.001	0.122	0.560
KLKB1	P03952	Olink	28	5	14	<0.001	0.202	0.065
SUOX	P51687	Olink	27	19	5	<0.001	<0.001	0.409
NEFL	P07196	Olink	25	13	14	<0.001	0.006	0.020
OPN		IA	25	4	5	<0.001	0.295	0.530
LIPR1	P54315	Olink	24	8	18	0.829	0.706	0.096
FLT3L	P49771	Olink	23	9	9	0.211	0.097	0.526
AHNAK	Q09666	Olink	23	8	36	<0.001	<0.001	0.013
MXRA8	Q9BRK3	Olink	19	4	5	<0.001	0.827	0.058
ADAMTS16	Q8TE57	Olink	19	3	9	<0.001	0.857	0.484
THBS1		IA	17	15	9	0.127	0.389	0.251
PPY	P01298	Olink	17	4	18	<0.001	0.030	0.743
REG4	Q9BYZ8	Olink	15	3	5	<0.001	0.900	0.292
KIRREL2	Q6UWL6	Olink	13	7	5	0.320	0.730	0.907
IL7R	P16871	Olink	13	5	9	<0.001	0.669	0.854
KLK10	O43240	Olink	13	4	18	0.501	0.431	0.018
TIMP2	P16035	Olink	12	16	14	0.050	0.019	0.362
VEGFC	P49767	Olink	12	12	5	0.763	0.320	0.560
SDF1	P48061	Olink	9	1	9	0.484	0.040	0.829
CA153		IA	4	11	0	0.698	0.360	0.290

a CP = Chronic pancreatitis, NOD = New-onset diabetes, Target = HRI, HC, IPMN controls and stage I or II PDAC cases, spe = specificity, IA = Immunoassay.

b The sensitivity for each marker at 95–96% specificity is shown in the target (*n* = 208), CP (*n* = 125), and NOD (*n* = 71) population.

In the CP population, most of the 41 markers exhibited low sensitivity. CA19-9 (40%), MUC13 (36%), MERTK (32%), SEMA7A (27%), and PLA2G1B (18%) had significant *p*-values, although they showed low to medium sensitivity. Similarly, in the NOD population, the sensitivities of CA19-9 (41%), MERTK (23%), CPB1 (23%), NEFL (14%), AHNAK (36%), and KLK10 (18%) had significant *p*-values.

### Synergistic Effects between Biomarker Candidates

**Figure 2:**
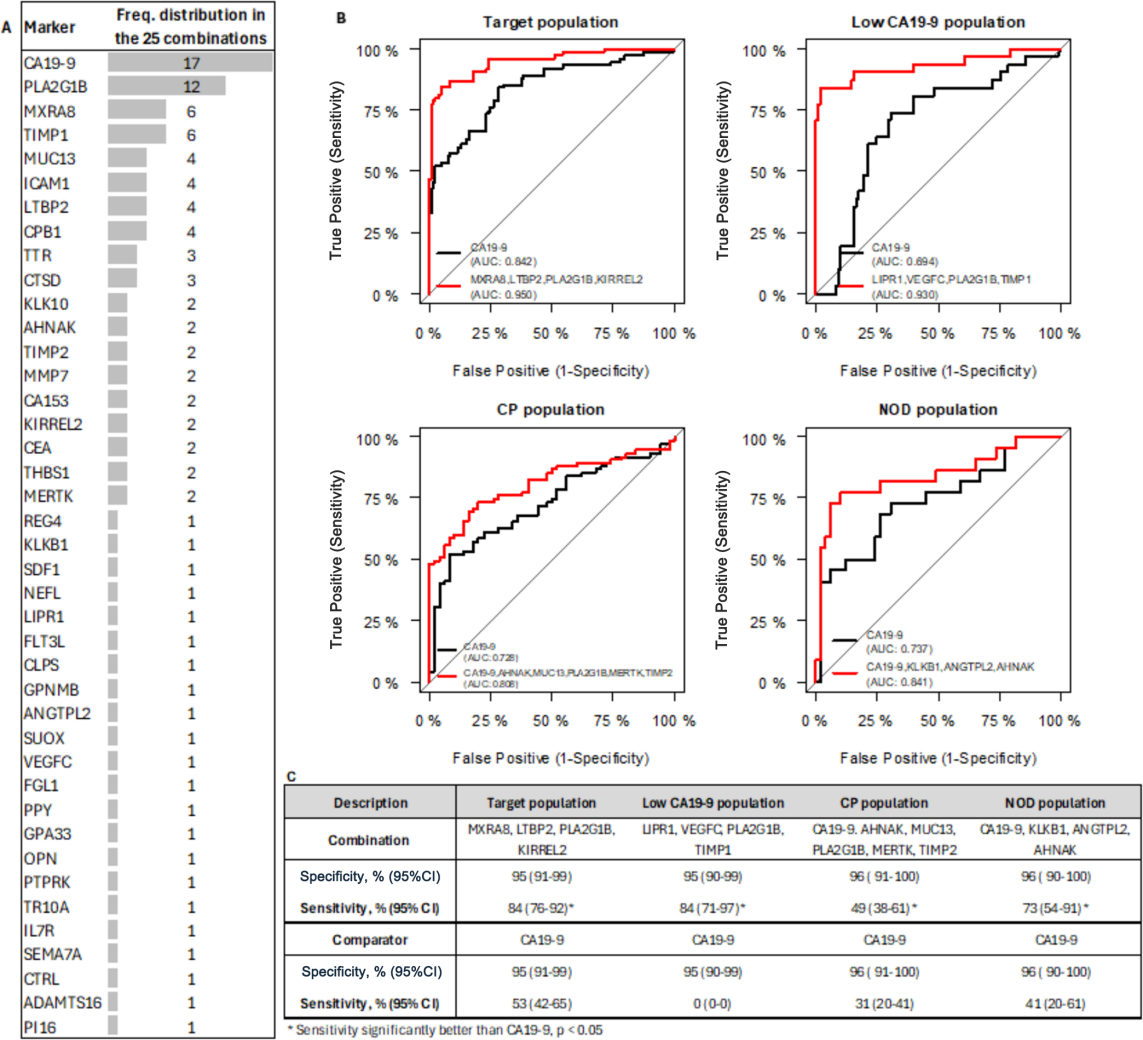
(A) Frequency distribution of the 41 markers in the 25 combinations, (B) ROC of the best combination (red) and CA19-9 (black) in the different populations, and (C) clinical performance at roughly 95% specificity of the best combinations compared to CA19-9 in the different subpopulations.

In the next step, biomarker candidates were combined to evaluate potential synergistic effects between individual protein markers. A total of 25 combinations of 4–6 markers out of the 41 markers were selected, based on their higher sensitivity when compared to CA19-9 alone at 95% specificity in the target, Low CA19-9, CP, or NOD population. The marker combinations and their clinical performance are listed in Table S4. The clinical performances of the best combinations in the different subpopulations are presented including the respective ROCs in Figure 2B.

At an equal specificity of 95%, nearly all combinations showed higher PDAC detection rates than CA19-9 in the target population, with sensitivities ranging from 60 to 84% compared to 53% sensitivity for CA19-9 alone, with the best combination being composed of MXRA8, LTBP2, PLA2G1B, and KIRREL2. All combinations show significantly better performance in detecting PDAC than CA19-9 in the low CA19-9 population, with LIPR1, VEGFC, PLA2G1B, and TIMP1 being the best combination, with a sensitivity of 84% at 95% sensitivity. Three and seven combinations in the CP and in the NOD population, respectively, demonstrated higher sensitivity than CA19-9 alone. The performance of the best combination (CA19-9, AHNAK, MUC13, PLA2G1B, MERTK, TIMP2) in the CP population was only slightly but still statistically significantly higher than that of CA19-9, with a sensitivity of 49% compared to 31% sensitivity for CA19-9 alone. In contrast, the improvement in PDAC detection of the best combination (CA19-9, KLKB1, ANGTPL2, AHNAK) in the NOD subpopulation was significant, with a sensitivity of 73% compared to 41% sensitivity for CA19-9 alone.

### IMMray PanCan-d Test

A subset of the Target population composed of 75 PDACs (36 stage I, 39 stage II), 50 healthy controls, and 47 high-risk individuals was analyzed using the formerly commercially available IMMray PanCan-d test and compared to CA19-9 alone (Figure S1). The comparison was performed using a single cutoff of 37 for CA19-9^26^ and 0.281 for the IMMray PanCan-d test without omitting borderline results.^10^ There was no significant difference in terms of sensitivity or specificity between the two tests: IMMray PanCan-d showed a sensitivity of 66% [55–77%] compared to 60% [49–72%] for CA19-9 alone (*p* = 0.157) and a specificity of 94% [89–99%] compared to 92% [86–97%] (*p* = 0.317). Importantly, multiple novel biomarker combinations discovered in this study showed higher clinical performance when compared to CA19-9 as shown above.

## Discussion

In this study, patient samples were analyzed using either targeted immunoassays or Olink PEA platform, covering approximately 3000 different proteins. The Olink platform covers proteins across a wide range of indications, including cardiometabolic, inflammation, neurology, and oncology. In contrast, the 27 targeted immunoassays consist of highly selected proteins related to pancreatic cancer according to the literature search.

Most of the measurements were robust and suitable for further analysis. However, roughly 20% of the Olink measurements required imputation using random forest techniques due to 0–20% data missing. In contrast, there were no missing data for the 27 immunoassays analyzed. For 16 proteins, both the Olink and targeted immunoassay data were available. More than 80% (13 out of 16) showed a medium or high correlation between the two assay formats. This is remarkable and certainly partly explained by the antibody-based detection used in both parts. Only for three assays was no correlation found. This certainly is encouraging for the future development of targeted immunoassays for all potential biomarker candidates for clinical application.

Finally, from this comprehensive proteomic data set resulting from the measurement of 329 well-characterized clinical samples, 41 unique proteins emerged as promising biomarker candidates for identifying patients with PDAC. Overall, the biomarkers identified are intricately involved in various biological pathways that contribute to pancreatic cancer progression including pathways associated with tumor progression, inflammation, and matrix remodeling. There is a notable trend of these proteins influencing one another’s roles in promoting or inhibiting tumorigenesis. Moreover, various combinations demonstrated significantly superior performance compared to that of the CA19-9 assay. The number of markers included in the combinations was limited to a maximum of 6 parameters to (1) avoid overfitting and (2) make it possible to transfer the findings into a clinical application that is commercially viable.

The clinical performance presented here warrants both the low false-positive rate (less than 5%) and the high sensitivity of PDAC (84%) needed for a PDAC screening test in the target population. The clinical data presented here for the best-performing signature composed of MXRA8, LTBP2, PLA2G1B, and KIRREL2 showed higher sensitivity (84%) and specificity (95%) compared to both CA19-9 (60%/92%) and the formerly commercially available IMMray PanCan-d test (66%/94%).

Despite these promising findings, the study has several limitations: first, the small number of patients in the different subgroups results in low statistical power, leading to wide confidence intervals in the clinical performance estimates. Additionally, the prevalence of PDAC in the study cohort does not reflect its prevalence in a real-world setting. The artificial composition of the cohort led to an overrepresentation of certain patient subgroups, such as those with CP. Furthermore, the NOD and CP population analyses were exploratory, as they are not powered to draw final conclusions. Therefore, the study cannot be used to evaluate the exact clinical performance of the biomarkers or combinations but rather as a discovery study for identifying new markers associated with PDAC.

A further limitation is the fact that all samples from the NOD population were collected at a single site, introducing a potential site bias that cannot be excluded. A final limitation is that the racial and ethnic compositions of the study participants do not reflect the diversity of the US population. Consequently, extrapolation of the findings to ethnicities other than Caucasians should be done with caution.

Larger, prospective multicenter studies are ongoing to further validate the findings presented here. Ultimately, the method presented is intended to be used as an aid in detecting PDAC among patients at risk of pancreatic cancer.

## Conclusions

Machine learning combined 2261 biomarkers into 4- to 6-plex signatures to distinguish PDAC from controls. These signatures significantly outperformed CA19-9, with an AUC of 0.95 and 84% sensitivity at 95% specificity, compared to CA19-9’s AUC of 0.84 and a sensitivity of 53%. A subset of 41 biomarkers can now be transitioned to further clinical validation in an independent set of PDAC and high-risk control samples using targeted immunoassays. This new multianalyte blood test could improve the early detection of PDAC in high-risk individuals.

## Data Availability

All data that support the findings of this study are provided in Table S5.

## Supporting Information Available

Performance of PanCan-d IMMray compared to CA19-9 (Figure S1); origin of the patient samples according to site and country (Table S1); Olink protein list with matched gene identifier (Table S2); list of immunoassay components provider (Table S3); and clinical performance of the 25 marker combinations in the subpopulations (Table S4) (PDF)
Data of all protein measurements including diagnosis of patient samples (Table S5) and additional data (ZIP)

## Abbreviations

PDAC: pancreatic ductal adenocarcinoma
PEA: Proximity Extension Explore Assay
ELISA: enzyme-linked immunosorbent assay
HC: healthy controls
CP: chronic pancreatitis
IPMN: intraductal papillary mucinous neoplasm
NOD: new-onset diabetes
HRI: high-risk individuals
