# Supplementary material for: Biomarker Discovery for Early Detection of Pancreatic Ductal Adenocarcinoma (PDAC) Using Multiplex Proteomics Technology
Source: J Proteome Res. 2024 Dec 19;24(1):315–22. doi: 10.1021/acs.jproteome.4c00752 (PMC11705213; doi:10.1021/acs.jproteome.4c00752)
Supplement: Supplementary file 1 — pr4c00752_si_001.pdf [file pr4c00752_si_001.pdf]

# Supporting Information

## Biomarker Discovery for Early Detection of Pancreatic Ductal Adenocarcinoma (PDAC) using Multiplex Proteomics Technology

*Alcibiade Athanasiou[1)], Natasha Kureshi[2)], Anja Wittig[1)], Maria Sterner[3)], Ramy Huber[1)], Norma A. Palma[2)], Thomas King[2)], Ralph Schiess[1)*]*

1) Proteomedix AG, Wagistrasse 23, CH-8952 Schlieren, Switzerland.
2) Immunovia Inc., 26 Forest Street, Suite 110, Marlborough, MA 01752, USA
3) Immunovia AB, Medicon Village, Scheelevägen 8, SE-223 63 Lund, Sweden.

* *schiess@proteomedix.com*

## Table of Content: Supplementary Tables and Figures

- **Supplementary Figure S1:** Performance of PanCan-d IMMray compared to CA19-9.
- **Supplementary Table S1:** Origin of the patient samples according to site and country.
- **Supplementary Table S2:** Olink protein list with matched gene identifier.
- **Supplementary Table S3:** List of immunoassay components provider
- **Supplementary Table S4**: Clinical performance of the 25 marker combinations in the sub-populations
- **Supplementary Table S5:** Data of all protein measurements including diagnosis of patient samples

**Supplementary Figure S1**: Performance of PanCan-d IMMray compared to CA19-9. (A) Population description, (B) clinical performance, (C) ROC of CA19-9 (black) and IMMray (red).

**A**

| Parameter | Controls HC | Controls HRI | Cases High CA19-9 | Cases Low CA19-9 |
|---|---|---|---|---|
| n | 48 | 47 | 44 | 29 |
| Age, y | 58 (53,63) | 55 (48,60) | 73 (65,78) | 62 (49,67) |
| CA19-9, U/mL | 7 (5,11) | 9 (5,22) | 132 (75,256) | 11 (0,16) |
| IMMay, DV | 1.64 (1.13,1.99) | 1.45 (0.98,1.87) | -0.54(-1.15,-0.29) | 0.42 (-0.45,0.8) |
| Male, % | 58 | 47 | 45 | 48 |
| Female, % | 42 | 53 | 55 | 52 |

**B**

| Parameter | CA19-9 | PanCan-D IMMray | p.value. |
|---|---|---|---|
| cutoff | 37 | 0.281 | - |
| AUC | 0.870 (0.813-0.928) | 0.878 (0.821-0.935) | 0.653 |
| Sensitivity | 60% (49-72%) | 66% (55-77%) | 0.157 |
| Specificity | 92% (86-97%) | 94% (89-99%) | 0.317 |
| NPV | 75% (67-83%) | 78% (74-86%) | 0.113 |
| PPV | 85% (75-94%) | 89% (84-97%) | 0.214 |

**C**

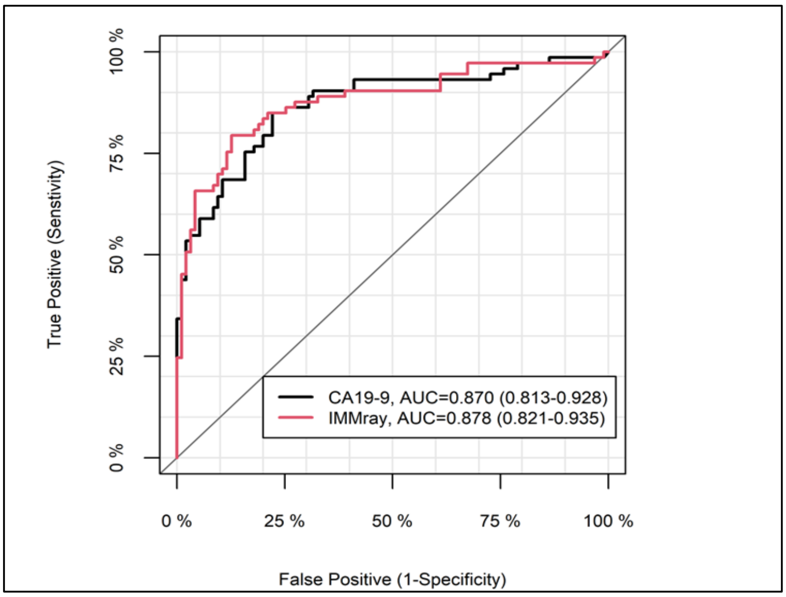

**Supplementary Table S1**: Origin of the patient samples according site and country

| Site | Country | Controls HC | Controls CP | Controls HRI | Controls IPMN | Controls NOD | PDAC NOD | PDAC Stage 1 | PDAC Stage 2 | Total |
|---|---|---|---|---|---|---|---|---|---|---|
| Hvidovre | DEN | - | 24 | - | - | - | - | - | - | 24 |
| ICO Spain | ESP | - | - | 8 | - | - | - | - | - | 8 |
| Ramón y Cajal | ESP | - | - | - | - | - | - | 3 | 3 | 6 |
| Santiago de Compostela | ESP | - | - | 10 | - | - | - | - | - | 10 |
| Helsinki | FIN | - | - | - | - | - | - | 4 | 7 | 11 |
| Erlangen | GER | - | 26 | - | 26 | - | - | 6 | 3 | 61 |
| Linköping | SWE | - | - | 11 | - | - | - | - | - | 11 |
| Ljungby | SWE | 14 | - | - | - | - | - | - | - | 14 |
| LUDC | SWE | - | - | - | - | 50 | 44 | - | - | 94 |
| Sahlgrenska | SWE | - | - | 18 | 10 | - | - | 13 | 20 | 61 |
| Varberg | SWE | 17 | - | - | - | - | - | - | - | 17 |
| Växjö | SWE | 19 | - | - | - | - | - | - | - | 19 |
| Pittsburgh | USA | - | - | - | - | - | - | 10 | 6 | 16 |
| **Total** | - | 50 | 50 | 47 | 36 | 50 | 44 | 36 | 39 | 352 |

**Supplementary Table S2**: List of the 2250 markers measured using Olink technology

| | Gene | Protein | | Gene | Protein | | Gene | Protein | | Gene | Protein |
|---|---|---|---|---|---|---|---|---|---|---|---|
| 1 | A0FGR8 | ESYT2_HUMAN | 79 | O15031 | PLXB2_HUMAN | 157 | O60447 | EVI5_HUMAN | 235 | O76076 | CCN5_HUMAN |
| 2 | A1E959 | ODAM_HUMAN | 80 | O15117 | FYB1_HUMAN | 158 | O60449 | LY75_HUMAN | 236 | O76096 | CYTF_HUMAN |
| 3 | A1KZ92 | PXDNL_HUMAN | 81 | O15123 | ANGP2_HUMAN | 159 | O60462 | NRP2_HUMAN | 237 | O77932 | DXO_HUMAN |
| 4 | A1L4H1 | SRCRL_HUMAN | 82 | O15164 | TIF1A_HUMAN | 160 | O60469 | DSCAM_HUMAN | 238 | O94760 | DDAH1_HUMAN |
| 5 | A4D1B5 | GSAP_HUMAN | 83 | O15169 | AXIN1_HUMAN | 161 | O60476 | MA1A2_HUMAN | 239 | O94779 | CNTN5_HUMAN |
| 6 | A6BM72 | MEG11_HUMAN | 84 | O15182 | CETN3_HUMAN | 162 | O60496 | DOK2_HUMAN | 240 | O94813 | SLIT2_HUMAN |
| 7 | A6NC86 | PINLY_HUMAN | 85 | O15197 | EPHB6_HUMAN | 163 | O60500 | NPHN_HUMAN | 241 | O94830 | DDHD2_HUMAN |
| 8 | A6NFN3 | RFOX3_HUMAN | 86 | O15213 | WDR46_HUMAN | 164 | O60502 | OGA_HUMAN | 242 | O94856 | NFASC_HUMAN |
| 9 | A6NI73 | LIRA5_HUMAN | 87 | O15232 | MATN3_HUMAN | 165 | O60542 | PSPN_HUMAN | 243 | O94903 | PLPHP_HUMAN |
| 10 | A6NM11 | L37A2_HUMAN | 88 | O15240 | VGF_HUMAN | 166 | O60575 | ISK4_HUMAN | 244 | O94907 | DKK1_HUMAN |
| 11 | A8MVW0 | F1712_HUMAN | 89 | O15263 | DFB4A_HUMAN | 167 | O60609 | GFRA3_HUMAN | 245 | O94979 | SC31A_HUMAN |
| 12 | A8MVW5 | HECA2_HUMAN | 90 | O15264 | MK13_HUMAN | 168 | O60662 | KLH41_HUMAN | 246 | O94986 | CE152_HUMAN |
| 13 | B1AKI9 | ISM1_HUMAN | 91 | O15269 | SPTC1_HUMAN | 169 | O60664 | PLIN3_HUMAN | 247 | O94988 | FA13A_HUMAN |
| 14 | B2RUY7 | VWC2L_HUMAN | 92 | O15305 | PMM2_HUMAN | 170 | O60760 | HPGDS_HUMAN | 248 | O94992 | HEXI1_HUMAN |
| 15 | B6SEH8 | ERVV1_HUMAN | 93 | O15335 | CHAD_HUMAN | 171 | O60763 | USO1_HUMAN | 249 | O95157 | NXPH3_HUMAN |
| 16 | I3L3R5 | CCER2_HUMAN | 94 | O15354 | GPR37_HUMAN | 172 | O60825 | F262_HUMAN | 250 | O95166 | GBRAP_HUMAN |
| 17 | O00161 | SNP23_HUMAN | 95 | O15357 | SHIP2_HUMAN | 173 | O60869 | EDF1_HUMAN | 251 | O95183 | VAMP5_HUMAN |
| 18 | O00175 | CCL24_HUMAN | 96 | O15389 | SIGL5_HUMAN | 174 | O60880 | SH21A_HUMAN | 252 | O95196 | CSPG5_HUMAN |
| 19 | O00182 | LEG9_HUMAN | 97 | O15394 | NCAM2_HUMAN | 175 | O60884 | DNJA2_HUMAN | 253 | O95202 | LETM1_HUMAN |
| 20 | O00186 | STXB3_HUMAN | 98 | O15400 | STX7_HUMAN | 176 | O60890 | OPHN1_HUMAN | 254 | O95256 | I18RA_HUMAN |
| 21 | O00194 | RB27B_HUMAN | 99 | O15444 | CCL25_HUMAN | 177 | O60907 | TBL1X_HUMAN | 255 | O95274 | LYPD3_HUMAN |
| 22 | O00203 | AP3B1_HUMAN | 100 | O15455 | TLR3_HUMAN | 178 | O60911 | CATL2_HUMAN | 256 | O95295 | SNAPN_HUMAN |
| 23 | O00206 | TLR4_HUMAN | 101 | O15467 | CCL16_HUMAN | 179 | O60934 | NBN_HUMAN | 257 | O95379 | TFIP8_HUMAN |
| 24 | O00214 | LEG8_HUMAN | 102 | O15496 | PA2GX_HUMAN | 180 | O60941 | DTNB_HUMAN | 258 | O95388 | CCN4_HUMAN |
| 25 | O00220 | TR10A_HUMAN | 103 | O43155 | FLRT2_HUMAN | 181 | O75015 | FCG3B_HUMAN | 259 | O95393 | BMP10_HUMAN |
| 26 | O00221 | IKBE_HUMAN | 104 | O43184 | ADA12_HUMAN | 182 | O75023 | LIRB5_HUMAN | 260 | O95407 | TNF6B_HUMAN |
| 27 | O00233 | PSMD9_HUMAN | 105 | O43240 | KLK10_HUMAN | 183 | O75054 | IGSF3_HUMAN | 261 | O95429 | BAG4_HUMAN |
| 28 | O00241 | SIRB1_HUMAN | 106 | O43278 | SPIT1_HUMAN | 184 | O75061 | AUXI_HUMAN | 262 | O95445 | APOM_HUMAN |
| 29 | O00244 | ATOX1_HUMAN | 107 | O43280 | TREA_HUMAN | 185 | O75071 | EFC14_HUMAN | 263 | O95466 | FMNL1_HUMAN |
| 30 | O00253 | AGRP_HUMAN | 108 | O43290 | SNUT1_HUMAN | 186 | O75077 | ADA23_HUMAN | 264 | O95467 | GNAS3_HUMAN |
| 31 | O00273 | DFFA_HUMAN | 109 | O43291 | SPIT2_HUMAN | 187 | O75121 | MFA3L_HUMAN | 265 | O95497 | VNN1_HUMAN |
| 32 | O00292 | LFTY2_HUMAN | 110 | O43312 | MTSS1_HUMAN | 188 | O75144 | ICOSL_HUMAN | 266 | O95498 | VNN2_HUMAN |
| 33 | O00300 | TR11B_HUMAN | 111 | O43320 | FGF16_HUMAN | 189 | O75146 | HIP1R_HUMAN | 267 | O95502 | NPTXR_HUMAN |
| 34 | O00308 | WWP2_HUMAN | 112 | O43399 | TPD54_HUMAN | 190 | O75154 | RFIP3_HUMAN | 268 | O95544 | NADK_HUMAN |
| 35 | O00339 | MATN2_HUMAN | 113 | O43405 | COCH_HUMAN | 191 | O75167 | PHAR2_HUMAN | 269 | O95630 | STABP_HUMAN |
| 36 | O00391 | QSOX1_HUMAN | 114 | O43422 | P52K_HUMAN | 192 | O75173 | ATS4_HUMAN | 270 | O95633 | FSTL3_HUMAN |
| 37 | O00451 | GFRA2_HUMAN | 115 | O43432 | IF4G3_HUMAN | 193 | O75190 | DNJB6_HUMAN | 271 | O95644 | NFAC1_HUMAN |
| 38 | O00468 | AGRIN_HUMAN | 116 | O43464 | HTRA2_HUMAN | 194 | O75223 | GGCT_HUMAN | 272 | O95684 | CEP43_HUMAN |
| 39 | O00533 | NCHL1_HUMAN | 117 | O43474 | KLF4_HUMAN | 195 | O75312 | ZPR1_HUMAN | 273 | O95721 | SNP29_HUMAN |
| 40 | O00548 | DLL1_HUMAN | 118 | O43493 | TGON2_HUMAN | 196 | O75326 | SEM7A_HUMAN | 274 | O95727 | CRTAM_HUMAN |
| 41 | O00559 | RCAS1_HUMAN | 119 | O43504 | LTOR5_HUMAN | 197 | O75339 | CILP1_HUMAN | 275 | O95750 | FGF19_HUMAN |
| 42 | O00584 | RNT2_HUMAN | 120 | O43505 | B4GA1_HUMAN | 198 | O75340 | PDCD6_HUMAN | 276 | O95786 | RIGI_HUMAN |
| 43 | O00585 | CCL21_HUMAN | 121 | O43508 | TNF12_HUMAN | 199 | O75347 | TBCA_HUMAN | 277 | O95793 | STAU1_HUMAN |
| 44 | O00592 | PODXL_HUMAN | 122 | O43557 | TNF14_HUMAN | 200 | O75348 | VATG1_HUMAN | 278 | O95817 | BAG3_HUMAN |
| 45 | O00602 | FCN1_HUMAN | 123 | O43561 | LAT_HUMAN | 201 | O75351 | VPS4B_HUMAN | 279 | O95825 | QORL1_HUMAN |
| 46 | O00622 | CCN1_HUMAN | 124 | O43570 | CAH12_HUMAN | 202 | O75354 | ENTP6_HUMAN | 280 | O95831 | AIFM1_HUMAN |
| 47 | O00626 | CCL22_HUMAN | 125 | O43583 | DENR_HUMAN | 203 | O75356 | ENTP5_HUMAN | 281 | O95841 | ANGL1_HUMAN |
| 48 | O00748 | EST2_HUMAN | 126 | O43597 | SPY2_HUMAN | 204 | O75365 | TP4A3_HUMAN | 282 | O95866 | G6B_HUMAN |
| 49 | O14523 | C2C2L_HUMAN | 127 | O43598 | DNPH1_HUMAN | 205 | O75380 | NDUS6_HUMAN | 283 | O95954 | FTCD_HUMAN |
| 50 | O14558 | HSPB6_HUMAN | 128 | O43639 | NCK2_HUMAN | 206 | O75427 | LRCH4_HUMAN | 284 | O95965 | ITGBL_HUMAN |
| 51 | O14594 | NCAN_HUMAN | 129 | O43653 | PSCA_HUMAN | 207 | O75460 | ERN1_HUMAN | 285 | O95971 | BY55_HUMAN |
| 52 | O14618 | CCS_HUMAN | 130 | O43665 | RGS10_HUMAN | 208 | O75462 | CRLF1_HUMAN | 286 | O95980 | RECK_HUMAN |
| 53 | O14625 | CXL11_HUMAN | 131 | O43681 | GET3_HUMAN | 209 | O75475 | PSIP1_HUMAN | 287 | O95994 | AGR2_HUMAN |
| 54 | O14662 | STX16_HUMAN | 132 | O43699 | SIGL6_HUMAN | 210 | O75493 | CAH11_HUMAN | 288 | O95998 | I18BP_HUMAN |
| 55 | O14717 | TRDMT_HUMAN | 133 | O43715 | TRIA1_HUMAN | 211 | O75506 | HSBP1_HUMAN | 289 | O96007 | MOC2B_HUMAN |
| 56 | O14737 | PDCD5_HUMAN | 134 | O43736 | ITM2A_HUMAN | 212 | O75509 | TNR21_HUMAN | 290 | P00167 | CYB5_HUMAN |
| 57 | O14745 | NHRF1_HUMAN | 135 | O43752 | STX6_HUMAN | 213 | O75521 | ECI2_HUMAN | 291 | P00325 | ADH1B_HUMAN |
| 58 | O14763 | TR10B_HUMAN | 136 | O43805 | SSNA1_HUMAN | 214 | O75563 | SKAP2_HUMAN | 292 | P00352 | AL1A1_HUMAN |
| 59 | O14773 | TPP1_HUMAN | 137 | O43854 | EDIL3_HUMAN | 215 | O75569 | PRKRA_HUMAN | 293 | P00390 | GSHR_HUMAN |
| 60 | O14786 | NRP1_HUMAN | 138 | O43866 | CD5L_HUMAN | 216 | O75592 | MYCB2_HUMAN | 294 | P00441 | SODC_HUMAN |
| 61 | O14788 | TNF11_HUMAN | 139 | O43895 | XPP2_HUMAN | 217 | O75629 | CREG1_HUMAN | 295 | P00519 | ABL1_HUMAN |
| 62 | O14791 | APOL1_HUMAN | 140 | O43896 | KIF1C_HUMAN | 218 | O75665 | OFD1_HUMAN | 296 | P00533 | EGFR_HUMAN |
| 63 | O14793 | GDF8_HUMAN | 141 | O43903 | GAS2_HUMAN | 219 | O75695 | XRP2_HUMAN | 297 | P00568 | KAD1_HUMAN |
| 64 | O14798 | TR10C_HUMAN | 142 | O43915 | VEGFD_HUMAN | 220 | O75711 | SCRG1_HUMAN | 298 | P00734 | THRB_HUMAN |
| 65 | O14828 | SCAM3_HUMAN | 143 | O43927 | CXL13_HUMAN | 221 | O75781 | PALM_HUMAN | 299 | P00736 | C1R_HUMAN |
| 66 | O14836 | TR13B_HUMAN | 144 | O60218 | AK1BA_HUMAN | 222 | O75787 | RENR_HUMAN | 300 | P00740 | FA9_HUMAN |
| 67 | O14867 | BACH1_HUMAN | 145 | O60232 | ZNRD2_HUMAN | 223 | O75791 | GRAP2_HUMAN | 301 | P00742 | FA10_HUMAN |
| 68 | O14879 | IFIT3_HUMAN | 146 | O60234 | GMFG_HUMAN | 224 | O75792 | RNH2A_HUMAN | 302 | P00746 | CFAD_HUMAN |
| 69 | O14904 | WNT9A_HUMAN | 147 | O60235 | TM11D_HUMAN | 225 | O75830 | SPI2_HUMAN | 303 | P00747 | PLMN_HUMAN |
| 70 | O14917 | PCD17_HUMAN | 148 | O60237 | MYPT2_HUMAN | 226 | O75843 | AP1G2_HUMAN | 304 | P00748 | FA12_HUMAN |
| 71 | O14933 | UB2L6_HUMAN | 149 | O60238 | BNI3L_HUMAN | 227 | O75882 | ATRN_HUMAN | 305 | P00749 | UROK_HUMAN |
| 72 | O14944 | EREG_HUMAN | 150 | O60240 | PLIN1_HUMAN | 228 | O75888 | TNF13_HUMAN | 306 | P00750 | TPA_HUMAN |
| 73 | O14960 | LECT2_HUMAN | 151 | O60242 | AGRB3_HUMAN | 229 | O75940 | SPF30_HUMAN | 307 | P00751 | CFAB_HUMAN |
| 74 | O14964 | HGS_HUMAN | 152 | O60243 | H6ST1_HUMAN | 230 | O76036 | NCTR1_HUMAN | 308 | P00797 | RENI_HUMAN |
| 75 | O14967 | CLGN_HUMAN | 153 | O60245 | PCDH7_HUMAN | 231 | O76038 | SEGN_HUMAN | 309 | P00813 | ADA_HUMAN |
| 76 | O14974 | MYPT1_HUMAN | 154 | O60259 | KLK8_HUMAN | 232 | O76061 | STC2_HUMAN | 310 | P00915 | CAH1_HUMAN |
| 77 | O15013 | ARHGA_HUMAN | 155 | O60279 | SUSD5_HUMAN | 233 | O76070 | SYUG_HUMAN | 311 | P00918 | CAH2_HUMAN |
| 78 | O15018 | PDZD2_HUMAN | 156 | O60437 | PEPL_HUMAN | 234 | O76074 | PDE5A_HUMAN | 312 | P00966 | ASSY_HUMAN |

| | Gene | Protein | | Gene | Protein | | Gene | Protein | | Gene | Protein |
|---|---|---|---|---|---|---|---|---|---|---|---|
| 313 | P00995 | ISK1_HUMAN | 391 | P04070 | PROC_HUMAN | 469 | P07320 | CRGD_HUMAN | 547 | P09769 | FGR_HUMAN |
| 314 | P01008 | ANT3_HUMAN | 392 | P04080 | CYTB_HUMAN | 470 | P07333 | CSF1R_HUMAN | 548 | P09871 | C1S_HUMAN |
| 315 | P01009 | A1AT_HUMAN | 393 | P04085 | PDGFA_HUMAN | 471 | P07339 | CATD_HUMAN | 549 | P09919 | CSF3_HUMAN |
| 316 | P01011 | AACT_HUMAN | 394 | P04090 | REL2_HUMAN | 472 | P07355 | ANXA2_HUMAN | 550 | P09923 | PPBI_HUMAN |
| 317 | P01019 | ANGT_HUMAN | 395 | P04114 | APOB_HUMAN | 473 | P07358 | CO8B_HUMAN | 551 | P09958 | FURIN_HUMAN |
| 318 | P01024 | CO3_HUMAN | 396 | P04118 | COL_HUMAN | 474 | P07359 | GP1BA_HUMAN | 552 | P09960 | LKHA4_HUMAN |
| 319 | P01031 | CO5_HUMAN | 397 | P04141 | CSF2_HUMAN | 475 | P07451 | CAH3_HUMAN | 553 | P0C862 | C1T9A_HUMAN |
| 320 | P01033 | TIMP1_HUMAN | 398 | P04155 | TFF1_HUMAN | 476 | P07478 | TRY2_HUMAN | 554 | P0CG30 | GSTT2_HUMAN |
| 321 | P01034 | CYTC_HUMAN | 399 | P04179 | SODM_HUMAN | 477 | P07492 | GRP_HUMAN | 555 | P0CG37 | CFC1_HUMAN |
| 322 | P01037 | CYTN_HUMAN | 400 | P04180 | LCAT_HUMAN | 478 | P07585 | PGS2_HUMAN | 556 | P0DJD7 | PEPA4_HUMAN |
| 323 | P01112 | RASH_HUMAN | 401 | P04196 | HRG_HUMAN | 479 | P07602 | SAP_HUMAN | 557 | P0DMV8 | HS71A_HUMAN |
| 324 | P01127 | PDGFB_HUMAN | 402 | P04216 | THY1_HUMAN | 480 | P07711 | CATL1_HUMAN | 558 | P0DOY2 | IGLC2_HUMAN |
| 325 | P01130 | LDLR_HUMAN | 403 | P04217 | A1BG_HUMAN | 481 | P07741 | APT_HUMAN | 559 | P0DPI2 | GAL3A_HUMAN |
| 326 | P01133 | EGF_HUMAN | 404 | P04233 | HG2A_HUMAN | 482 | P07858 | CATB_HUMAN | 560 | P0DUB6 | AMY1A_HUMAN |
| 327 | P01135 | TGFA_HUMAN | 405 | P04275 | VWF_HUMAN | 483 | P07911 | UROM_HUMAN | 561 | P10082 | PYY_HUMAN |
| 328 | P01137 | TGFB1_HUMAN | 406 | P04278 | SHBG_HUMAN | 484 | P07942 | LAMB1_HUMAN | 562 | P10092 | CALCB_HUMAN |
| 329 | P01178 | NEU1_HUMAN | 407 | P04406 | G3P_HUMAN | 485 | P07947 | YES_HUMAN | 563 | P10145 | IL8_HUMAN |
| 330 | P01189 | COLI_HUMAN | 408 | P04439 | HLAA_HUMAN | 486 | P07948 | LYN_HUMAN | 564 | P10147 | CCL3_HUMAN |
| 331 | P01210 | PENK_HUMAN | 409 | P04626 | ERBB2_HUMAN | 487 | P07949 | RET_HUMAN | 565 | P10398 | ARAF_HUMAN |
| 332 | P01222 | TSHB_HUMAN | 410 | P04637 | P53_HUMAN | 488 | P07998 | RNAS1_HUMAN | 566 | P10415 | BCL2_HUMAN |
| 333 | P01229 | LSHB_HUMAN | 411 | P04746 | AMYP_HUMAN | 489 | P08069 | IGF1R_HUMAN | 567 | P10451 | OSTP_HUMAN |
| 334 | P01236 | PRL_HUMAN | 412 | P04792 | HSPB1_HUMAN | 490 | P08118 | MSMB_HUMAN | 568 | P10586 | PTPRF_HUMAN |
| 335 | P01241 | SOMA_HUMAN | 413 | P04808 | REL1_HUMAN | 491 | P08134 | RHOC_HUMAN | 569 | P10599 | THIO_HUMAN |
| 336 | P01242 | SOM2_HUMAN | 414 | P05026 | AT1B1_HUMAN | 492 | P08138 | TNR16_HUMAN | 570 | P10643 | CO7_HUMAN |
| 337 | P01258 | CALC_HUMAN | 415 | P05060 | SCG1_HUMAN | 493 | P08174 | DAF_HUMAN | 571 | P10644 | KAP0_HUMAN |
| 338 | P01266 | THYG_HUMAN | 416 | P05067 | A4_HUMAN | 494 | P08185 | CBG_HUMAN | 572 | P10645 | CMGA_HUMAN |
| 339 | P01270 | PTHY_HUMAN | 417 | P05089 | ARGI1_HUMAN | 495 | P08217 | CEL2A_HUMAN | 573 | P10646 | TFPI1_HUMAN |
| 340 | P01275 | GLUC_HUMAN | 418 | P05090 | APOD_HUMAN | 496 | P08236 | BGLR_HUMAN | 574 | P10721 | KIT_HUMAN |
| 341 | P01298 | PAHO_HUMAN | 419 | P05107 | ITB2_HUMAN | 497 | P08254 | MMP3_HUMAN | 575 | P10746 | HEM4_HUMAN |
| 342 | P01303 | NPY_HUMAN | 420 | P05121 | PAI1_HUMAN | 498 | P08263 | GSTA1_HUMAN | 576 | P10747 | CD28_HUMAN |
| 343 | P01350 | GAST_HUMAN | 421 | P05154 | IPSP_HUMAN | 499 | P08294 | SODE_HUMAN | 577 | P10909 | CLUS_HUMAN |
| 344 | P01375 | TNFA_HUMAN | 422 | P05155 | IC1_HUMAN | 500 | P08319 | ADH4_HUMAN | 578 | P10912 | GHR_HUMAN |
| 345 | P01579 | IFNG_HUMAN | 423 | P05156 | CFAI_HUMAN | 501 | P08397 | HEM3_HUMAN | 579 | P11215 | ITAM_HUMAN |
| 346 | P01584 | IL1B_HUMAN | 424 | P05160 | F13B_HUMAN | 502 | P08473 | NEP_HUMAN | 580 | P11226 | MBL2_HUMAN |
| 347 | P01588 | EPO_HUMAN | 425 | P05164 | PERM_HUMAN | 503 | P08519 | APOA_HUMAN | 581 | P11234 | RALB_HUMAN |
| 348 | P01589 | IL2RA_HUMAN | 426 | P05187 | PPB1_HUMAN | 504 | P08571 | CD14_HUMAN | 582 | P11274 | BCR_HUMAN |
| 349 | P01591 | IGJ_HUMAN | 427 | P05231 | IL6_HUMAN | 505 | P08575 | PTPRC_HUMAN | 583 | P11279 | LAMP1_HUMAN |
| 350 | P01730 | CD4_HUMAN | 428 | P05305 | EDN1_HUMAN | 506 | P08579 | RU2B_HUMAN | 584 | P11310 | ACADM_HUMAN |
| 351 | P01732 | CD8A_HUMAN | 429 | P05362 | ICAM1_HUMAN | 507 | P08581 | MET_HUMAN | 585 | P11387 | TOP1_HUMAN |
| 352 | P01833 | PIGR_HUMAN | 430 | P05412 | JUN_HUMAN | 508 | P08582 | TRFM_HUMAN | 586 | P11532 | DMD_HUMAN |
| 353 | P01903 | DRA_HUMAN | 431 | P05413 | FABPH_HUMAN | 509 | P08590 | MYL3_HUMAN | 587 | P11684 | UTER_HUMAN |
| 354 | P02008 | HBAZ_HUMAN | 432 | P05451 | REG1A_HUMAN | 510 | P08603 | CFAH_HUMAN | 588 | P11717 | MPRI_HUMAN |
| 355 | P02144 | MYG_HUMAN | 433 | P05452 | TETN_HUMAN | 511 | P08648 | ITA5_HUMAN | 589 | P12034 | FGF5_HUMAN |
| 356 | P02452 | CO1A1_HUMAN | 434 | P05543 | THBG_HUMAN | 512 | P08670 | VIME_HUMAN | 590 | P12104 | FABPI_HUMAN |
| 357 | P02458 | CO2A1_HUMAN | 435 | P05546 | HEP2_HUMAN | 513 | P08697 | A2AP_HUMAN | 591 | P12111 | CO6A3_HUMAN |
| 358 | P02461 | CO3A1_HUMAN | 436 | P05556 | ITB1_HUMAN | 514 | P08709 | FA7_HUMAN | 592 | P12270 | TPR_HUMAN |
| 359 | P02462 | CO4A1_HUMAN | 437 | P05783 | K1C18_HUMAN | 515 | P08727 | K1C19_HUMAN | 593 | P12277 | KCRB_HUMAN |
| 360 | P02647 | APOA1_HUMAN | 438 | P05937 | CALB1_HUMAN | 516 | P08758 | ANXA5_HUMAN | 594 | P12318 | FCG2A_HUMAN |
| 361 | P02649 | APOE_HUMAN | 439 | P06127 | CD5_HUMAN | 517 | P08833 | IBP1_HUMAN | 595 | P12319 | FCERA_HUMAN |
| 362 | P02652 | APOA2_HUMAN | 440 | P06132 | DCUP_HUMAN | 518 | P08887 | IL6RA_HUMAN | 596 | P12429 | ANXA3_HUMAN |
| 363 | P02654 | APOC1_HUMAN | 441 | P06276 | CHLE_HUMAN | 519 | P08962 | CD63_HUMAN | 597 | P12532 | KCRU_HUMAN |
| 364 | P02730 | B3AT_HUMAN | 442 | P06280 | AGAL_HUMAN | 520 | P09093 | CEL3A_HUMAN | 598 | P12544 | GRAA_HUMAN |
| 365 | P02743 | SAMP_HUMAN | 443 | P06396 | GELS_HUMAN | 521 | P09104 | ENOG_HUMAN | 599 | P12644 | BMP4_HUMAN |
| 366 | P02745 | C1QA_HUMAN | 444 | P06401 | PRGR_HUMAN | 522 | P09105 | HBAT_HUMAN | 600 | P12724 | ECP_HUMAN |
| 367 | P02748 | CO9_HUMAN | 445 | P06681 | CO2_HUMAN | 523 | P09110 | THIK_HUMAN | 601 | P12821 | ACE_HUMAN |
| 368 | P02749 | APOH_HUMAN | 446 | P06727 | APOA4_HUMAN | 524 | P09172 | DOPO_HUMAN | 602 | P12829 | MYL4_HUMAN |
| 369 | P02750 | A2GL_HUMAN | 447 | P06729 | CD2_HUMAN | 525 | P09211 | GSTP1_HUMAN | 603 | P12830 | CADH1_HUMAN |
| 370 | P02751 | FINC_HUMAN | 448 | P06730 | IF4E_HUMAN | 526 | P09237 | MMP7_HUMAN | 604 | P12872 | MOTI_HUMAN |
| 371 | P02760 | AMBP_HUMAN | 449 | P06731 | CEAM5_HUMAN | 527 | P09238 | MMP10_HUMAN | 605 | P12931 | SRC_HUMAN |
| 372 | P02763 | A1AG1_HUMAN | 450 | P06733 | ENOA_HUMAN | 528 | P09326 | CD48_HUMAN | 606 | P12955 | PEPD_HUMAN |
| 373 | P02765 | FETUA_HUMAN | 451 | P06734 | FCER2_HUMAN | 529 | P09341 | GROA_HUMAN | 607 | P13232 | IL7_HUMAN |
| 374 | P02766 | TTHY_HUMAN | 452 | P06744 | G6PI_HUMAN | 530 | P09382 | LEG1_HUMAN | 608 | P13236 | CCL4_HUMAN |
| 375 | P02771 | FETA_HUMAN | 453 | P06748 | NPM_HUMAN | 531 | P09417 | DHPR_HUMAN | 609 | P13284 | GILT_HUMAN |
| 376 | P02774 | VTDB_HUMAN | 454 | P06756 | ITAV_HUMAN | 532 | P09455 | RET1_HUMAN | 610 | P13385 | TDGF1_HUMAN |
| 377 | P02775 | CXCL7_HUMAN | 455 | P06850 | CRF_HUMAN | 533 | P09466 | PAEP_HUMAN | 611 | P13473 | LAMP2_HUMAN |
| 378 | P02776 | PLF4_HUMAN | 456 | P06858 | LIPL_HUMAN | 534 | P09467 | F16P1_HUMAN | 612 | P13500 | CCL2_HUMAN |
| 379 | P02778 | CXL10_HUMAN | 457 | P06870 | KLK1_HUMAN | 535 | P09486 | SPRC_HUMAN | 613 | P13501 | CCL5_HUMAN |
| 380 | P02786 | TFR1_HUMAN | 458 | P07093 | GDN_HUMAN | 536 | P09496 | CLCA_HUMAN | 614 | P13521 | SCG2_HUMAN |
| 381 | P02787 | TRFE_HUMAN | 459 | P07098 | LIPF_HUMAN | 537 | P09525 | ANXA4_HUMAN | 615 | P13591 | NCAM1_HUMAN |
| 382 | P02818 | OSTCN_HUMAN | 460 | P07108 | ACBP_HUMAN | 538 | P09529 | INHBB_HUMAN | 616 | P13598 | ICAM2_HUMAN |
| 383 | P03950 | ANGI_HUMAN | 461 | P07148 | FABPL_HUMAN | 539 | P09543 | CN37_HUMAN | 617 | P13611 | CSPG2_HUMAN |
| 384 | P03951 | FA11_HUMAN | 462 | P07196 | NFL_HUMAN | 540 | P09564 | CD7_HUMAN | 618 | P13647 | K2C5_HUMAN |
| 385 | P03952 | KLKB1_HUMAN | 463 | P07204 | TRBM_HUMAN | 541 | P09601 | HMOX1_HUMAN | 619 | P13667 | PDIA4_HUMAN |
| 386 | P03956 | MMP1_HUMAN | 464 | P07225 | PROS_HUMAN | 542 | P09603 | CSF1_HUMAN | 620 | P13686 | PPA5_HUMAN |
| 387 | P04040 | CATA_HUMAN | 465 | P07237 | PDIA1_HUMAN | 543 | P09619 | PGFRB_HUMAN | 621 | P13688 | CEAM1_HUMAN |
| 388 | P04054 | PA21B_HUMAN | 466 | P07306 | ASGR1_HUMAN | 544 | P09668 | CATH_HUMAN | 622 | P13725 | ONCM_HUMAN |
| 389 | P04062 | GBA1_HUMAN | 467 | P07307 | ASGR2_HUMAN | 545 | P09693 | CD3G_HUMAN | 623 | P13726 | TF_HUMAN |
| 390 | P04066 | FUCO_HUMAN | 468 | P07311 | ACYP1_HUMAN | 546 | P09758 | TACD2_HUMAN | 624 | P13727 | PRG2_HUMAN |

| | Gene | Protein |
|---|---|---|
| 625 | P13796 | PLSL_HUMAN |
| 626 | P13807 | GYS1_HUMAN |
| 627 | P13861 | KAP2_HUMAN |
| 628 | P13929 | ENOB_HUMAN |
| 629 | P13987 | CD59_HUMAN |
| 630 | P14091 | CATE_HUMAN |
| 631 | P14136 | GFAP_HUMAN |
| 632 | P14151 | LYAM1_HUMAN |
| 633 | P14174 | MIF_HUMAN |
| 634 | P14207 | FOLR2_HUMAN |
| 635 | P14209 | CD99_HUMAN |
| 636 | P14210 | HGF_HUMAN |
| 637 | P14317 | HCLS1_HUMAN |
| 638 | P14384 | CBPM_HUMAN |
| 639 | P14543 | NID1_HUMAN |
| 640 | P14555 | PA2GA_HUMAN |
| 641 | P14778 | IL1R1_HUMAN |
| 642 | P14780 | MMP9_HUMAN |
| 643 | P14784 | IL2RB_HUMAN |
| 644 | P14854 | CX6B1_HUMAN |
| 645 | P14868 | SYDC_HUMAN |
| 646 | P14902 | I23O1_HUMAN |
| 647 | P15018 | LIF_HUMAN |
| 648 | P15085 | CBPA1_HUMAN |
| 649 | P15086 | CBPB1_HUMAN |
| 650 | P15090 | FABP4_HUMAN |
| 651 | P15121 | ALDR_HUMAN |
| 652 | P15144 | AMPN_HUMAN |
| 653 | P15151 | PVR_HUMAN |
| 654 | P15260 | INGR1_HUMAN |
| 655 | P15289 | ARSA_HUMAN |
| 656 | P15291 | B4GT1_HUMAN |
| 657 | P15311 | EZRI_HUMAN |
| 658 | P15328 | FOLR1_HUMAN |
| 659 | P15502 | ELN_HUMAN |
| 660 | P15509 | CSF2R_HUMAN |
| 661 | P15514 | AREG_HUMAN |
| 662 | P15529 | MCP_HUMAN |
| 663 | P15692 | VEGFA_HUMAN |
| 664 | P15848 | ARSB_HUMAN |
| 665 | P15907 | SIAT1_HUMAN |
| 666 | P16035 | TIMP2_HUMAN |
| 667 | P16109 | LYAM3_HUMAN |
| 668 | P16112 | PGCA_HUMAN |
| 669 | P16233 | LIPP_HUMAN |
| 670 | P16234 | PGFRA_HUMAN |
| 671 | P16278 | BGAL_HUMAN |
| 672 | P16284 | PECA1_HUMAN |
| 673 | P16422 | EPCAM_HUMAN |
| 674 | P16442 | BGAT_HUMAN |
| 675 | P16444 | DPEP1_HUMAN |
| 676 | P16455 | MGMT_HUMAN |
| 677 | P16562 | CRIS2_HUMAN |
| 678 | P16581 | LYAM2_HUMAN |
| 679 | P16671 | CD36_HUMAN |
| 680 | P16860 | ANFB_HUMAN |
| 681 | P16870 | CBPE_HUMAN |
| 682 | P16871 | IL7RA_HUMAN |
| 683 | P17050 | NAGAB_HUMAN |
| 684 | P17174 | AATC_HUMAN |
| 685 | P17181 | INAR1_HUMAN |
| 686 | P17301 | ITA2_HUMAN |
| 687 | P17405 | ASM_HUMAN |
| 688 | P17516 | AK1C4_HUMAN |
| 689 | P17538 | CTRB1_HUMAN |
| 690 | P17643 | TYRP1_HUMAN |
| 691 | P17676 | CEBPB_HUMAN |
| 692 | P17813 | EGLN_HUMAN |
| 693 | P17900 | SAP3_HUMAN |
| 694 | P17927 | CR1_HUMAN |
| 695 | P17931 | LEG3_HUMAN |
| 696 | P17936 | IBP3_HUMAN |
| 697 | P17948 | VGFR1_HUMAN |
| 698 | P18031 | PTN1_HUMAN |
| 699 | P18065 | IBP2_HUMAN |
| 700 | P18084 | ITB5_HUMAN |
| 701 | P18428 | LBP_HUMAN |
| 702 | P18510 | IL1RA_HUMAN |
| 703 | P18564 | ITB6_HUMAN |
| 704 | P18627 | LAG3_HUMAN |
| 705 | P18754 | RCC1_HUMAN |
| 706 | P18827 | SDC1_HUMAN |
| 707 | P19021 | AMD_HUMAN |
| 708 | P19022 | CADH2_HUMAN |
| 709 | P19075 | TSN8_HUMAN |
| 710 | P19256 | LFA3_HUMAN |
| 711 | P19320 | VCAM1_HUMAN |
| 712 | P19429 | TNNI3_HUMAN |
| 713 | P19438 | TNR1A_HUMAN |
| 714 | P19440 | GGT1_HUMAN |
| 715 | P19474 | RO52_HUMAN |
| 716 | P19525 | E2AK2_HUMAN |
| 717 | P19526 | FUT1_HUMAN |
| 718 | P19801 | AOC1_HUMAN |
| 719 | P19827 | ITIH1_HUMAN |
| 720 | P19838 | NFKB1_HUMAN |
| 721 | P19876 | CXCL3_HUMAN |
| 722 | P19878 | NCF2_HUMAN |
| 723 | P19883 | FST_HUMAN |
| 724 | P19957 | ELAF_HUMAN |
| 725 | P19961 | AMY2B_HUMAN |
| 726 | P19971 | TYPH_HUMAN |
| 727 | P20023 | CR2_HUMAN |
| 728 | P20042 | IF2B_HUMAN |
| 729 | P20061 | TCO1_HUMAN |
| 730 | P20062 | TCO2_HUMAN |
| 731 | P20138 | CD33_HUMAN |
| 732 | P20155 | ISK2_HUMAN |
| 733 | P20160 | CAP7_HUMAN |
| 734 | P20273 | CD22_HUMAN |
| 735 | P20333 | TNR1B_HUMAN |
| 736 | P20340 | RAB6A_HUMAN |
| 737 | P20472 | PRVA_HUMAN |
| 738 | P20645 | MPRD_HUMAN |
| 739 | P20700 | LMNB1_HUMAN |
| 740 | P20701 | ITAL_HUMAN |
| 741 | P20711 | DDC_HUMAN |
| 742 | P20718 | GRAH_HUMAN |
| 743 | P20742 | PZP_HUMAN |
| 744 | P20774 | MIME_HUMAN |
| 745 | P20783 | NTF3_HUMAN |
| 746 | P20807 | CAN3_HUMAN |
| 747 | P20809 | IL11_HUMAN |
| 748 | P20849 | CO9A1_HUMAN |
| 749 | P20851 | C4BPB_HUMAN |
| 750 | P20908 | CO5A1_HUMAN |
| 751 | P20916 | MAG_HUMAN |
| 752 | P20929 | NEBU_HUMAN |
| 753 | P21128 | ENDOU_HUMAN |
| 754 | P21217 | FUT3_HUMAN |
| 755 | P21246 | PTN_HUMAN |
| 756 | P21549 | AGT1_HUMAN |
| 757 | P21579 | SYT1_HUMAN |
| 758 | P21583 | SCF_HUMAN |
| 759 | P21589 | 5NTD_HUMAN |
| 760 | P21695 | GPDA_HUMAN |
| 761 | P21709 | EPHA1_HUMAN |
| 762 | P21741 | MK_HUMAN |
| 763 | P21754 | ZP3_HUMAN |
| 764 | P21757 | MSRE_HUMAN |
| 765 | P21781 | FGF7_HUMAN |
| 766 | P21802 | FGFR2_HUMAN |
| 767 | P21810 | PGS1_HUMAN |
| 768 | P21854 | CD72_HUMAN |
| 769 | P21860 | ERBB3_HUMAN |
| 770 | P21912 | SDHB_HUMAN |
| 771 | P21964 | COMT_HUMAN |
| 772 | P21980 | TGM2_HUMAN |
| 773 | P22079 | PERL_HUMAN |
| 774 | P22105 | TENX_HUMAN |
| 775 | P22223 | CADH3_HUMAN |
| 776 | P22301 | IL10_HUMAN |
| 777 | P22303 | ACES_HUMAN |
| 778 | P22304 | IDS_HUMAN |
| 779 | P22307 | SCP2_HUMAN |
| 780 | P22455 | FGFR4_HUMAN |
| 781 | P22466 | GALA_HUMAN |
| 782 | P22676 | CALB2_HUMAN |
| 783 | P22692 | IBP4_HUMAN |
| 784 | P22748 | CAH4_HUMAN |
| 785 | P22749 | GNLY_HUMAN |
| 786 | P22894 | MMP8_HUMAN |
| 787 | P22897 | MRC1_HUMAN |
| 788 | P23141 | EST1_HUMAN |
| 789 | P23229 | ITA6_HUMAN |
| 790 | P23276 | KELL_HUMAN |
| 791 | P23280 | CAH6_HUMAN |
| 792 | P23284 | PPIB_HUMAN |
| 793 | P23327 | SRCH_HUMAN |
| 794 | P23381 | SYWC_HUMAN |
| 795 | P23467 | PTPRB_HUMAN |
| 796 | P23471 | PTPRZ_HUMAN |
| 797 | P23515 | OMGP_HUMAN |
| 798 | P23526 | SAHH_HUMAN |
| 799 | P23560 | BDNF_HUMAN |
| 800 | P23582 | ANFC_HUMAN |
| 801 | P23588 | IF4B_HUMAN |
| 802 | P23919 | KTHY_HUMAN |
| 803 | P24001 | IL32_HUMAN |
| 804 | P24071 | FCAR_HUMAN |
| 805 | P24158 | PRTN3_HUMAN |
| 806 | P24387 | CRHBP_HUMAN |
| 807 | P24394 | IL4RA_HUMAN |
| 808 | P24530 | EDNRB_HUMAN |
| 809 | P24592 | IBP6_HUMAN |
| 810 | P24666 | PPAC_HUMAN |
| 811 | P24821 | TENA_HUMAN |
| 812 | P25116 | PAR1_HUMAN |
| 813 | P25440 | BRD2_HUMAN |
| 814 | P25445 | TNR6_HUMAN |
| 815 | P25685 | DNJB1_HUMAN |
| 816 | P25686 | DNJB2_HUMAN |
| 817 | P25774 | CATS_HUMAN |
| 818 | P25815 | S100P_HUMAN |
| 819 | P25942 | TNR5_HUMAN |
| 820 | P26010 | ITB7_HUMAN |
| 821 | P26022 | PTX3_HUMAN |
| 822 | P26378 | ELAV4_HUMAN |
| 823 | P26436 | ASPX_HUMAN |
| 824 | P26447 | S10A4_HUMAN |
| 825 | P26639 | SYTC_HUMAN |
| 826 | P26718 | NKG2D_HUMAN |
| 827 | P26842 | CD27_HUMAN |
| 828 | P26927 | HGFL_HUMAN |
| 829 | P26951 | IL3RA_HUMAN |
| 830 | P27169 | PON1_HUMAN |
| 831 | P27352 | IF_HUMAN |
| 832 | P27487 | DPP4_HUMAN |
| 833 | P27695 | APEX1_HUMAN |
| 834 | P27918 | PROP_HUMAN |
| 835 | P27930 | IL1R2_HUMAN |
| 836 | P28325 | CYTD_HUMAN |
| 837 | P28799 | GRN_HUMAN |
| 838 | P28827 | PTPRM_HUMAN |
| 839 | P28838 | AMPL_HUMAN |
| 840 | P28845 | DHI1_HUMAN |
| 841 | P28906 | CD34_HUMAN |
| 842 | P28907 | CD38_HUMAN |
| 843 | P28908 | TNR8_HUMAN |
| 844 | P29017 | CD1C_HUMAN |
| 845 | P29218 | IMPA1_HUMAN |
| 846 | P29279 | CCN2_HUMAN |
| 847 | P29317 | EPHA2_HUMAN |
| 848 | P29350 | PTN6_HUMAN |
| 849 | P29377 | S100G_HUMAN |
| 850 | P29459 | IL12A_HUMAN |
| 851 | P29460 | IL12B_HUMAN |
| 852 | P29474 | NOS3_HUMAN |
| 853 | P29475 | NOS1_HUMAN |
| 854 | P29536 | LMOD1_HUMAN |
| 855 | P29622 | KAIN_HUMAN |
| 856 | P29692 | EF1D_HUMAN |
| 857 | P29965 | CD40L_HUMAN |
| 858 | P30039 | PBLD_HUMAN |
| 859 | P30040 | ERP29_HUMAN |
| 860 | P30041 | PRDX6_HUMAN |
| 861 | P30043 | BLVRB_HUMAN |
| 862 | P30044 | PRDX5_HUMAN |
| 863 | P30046 | DOPD_HUMAN |
| 864 | P30047 | GFRP_HUMAN |
| 865 | P30084 | ECHM_HUMAN |
| 866 | P30086 | PEBP1_HUMAN |
| 867 | P30101 | PDIA3_HUMAN |
| 868 | P30203 | CD6_HUMAN |
| 869 | P30279 | CCND2_HUMAN |
| 870 | P30519 | HMOX2_HUMAN |
| 871 | P30530 | UFO_HUMAN |
| 872 | P30533 | AMRP_HUMAN |
| 873 | P30613 | KPYR_HUMAN |
| 874 | P30740 | ILEU_HUMAN |
| 875 | P31146 | COR1A_HUMAN |
| 876 | P31350 | RIR2_HUMAN |
| 877 | P31431 | SDC4_HUMAN |
| 878 | P31751 | AKT2_HUMAN |
| 879 | P31785 | IL2RG_HUMAN |
| 880 | P31948 | STIP1_HUMAN |
| 881 | P31949 | S10AB_HUMAN |
| 882 | P31994 | FCG2B_HUMAN |
| 883 | P31997 | CEAM8_HUMAN |
| 884 | P32004 | L1CAM_HUMAN |
| 885 | P32119 | PRDX2_HUMAN |
| 886 | P32320 | CDD_HUMAN |
| 887 | P32321 | DCTD_HUMAN |
| 888 | P32455 | GBP1_HUMAN |
| 889 | P32926 | DSG3_HUMAN |
| 890 | P32927 | IL3RB_HUMAN |
| 891 | P32942 | ICAM3_HUMAN |
| 892 | P32970 | CD70_HUMAN |
| 893 | P32971 | TNFL8_HUMAN |
| 894 | P33151 | CADH5_HUMAN |
| 895 | P33241 | LSP1_HUMAN |
| 896 | P33316 | DUT_HUMAN |
| 897 | P33681 | CD80_HUMAN |
| 898 | P33764 | S10A3_HUMAN |
| 899 | P34096 | RNAS4_HUMAN |
| 900 | P34130 | NTF4_HUMAN |
| 901 | P34896 | GLYC_HUMAN |
| 902 | P34947 | GRK5_HUMAN |
| 903 | P35052 | GPC1_HUMAN |
| 904 | P35070 | BTC_HUMAN |
| 905 | P35218 | CAH5A_HUMAN |
| 906 | P35228 | NOS2_HUMAN |
| 907 | P35237 | SPB6_HUMAN |
| 908 | P35247 | SFTPD_HUMAN |
| 909 | P35318 | ADML_HUMAN |
| 910 | P35442 | TSP2_HUMAN |
| 911 | P35443 | TSP4_HUMAN |
| 912 | P35475 | IDUA_HUMAN |
| 913 | P35520 | CBS_HUMAN |
| 914 | P35542 | SAA4_HUMAN |
| 915 | P35556 | FBN2_HUMAN |
| 916 | P35579 | MYH9_HUMAN |
| 917 | P35590 | TIE1_HUMAN |
| 918 | P35609 | ACTN2_HUMAN |
| 919 | P35611 | ADDA_HUMAN |
| 920 | P35613 | BASI_HUMAN |
| 921 | P35625 | TIMP3_HUMAN |
| 922 | P35637 | FUS_HUMAN |
| 923 | P35754 | GLRX1_HUMAN |
| 924 | P35916 | VGFR3_HUMAN |
| 925 | P35968 | VGFR2_HUMAN |
| 926 | P36222 | CH3L1_HUMAN |
| 927 | P36269 | GGT5_HUMAN |
| 928 | P36551 | HEM6_HUMAN |
| 929 | P36776 | LONM_HUMAN |
| 930 | P36873 | PP1G_HUMAN |
| 931 | P36888 | FLT3_HUMAN |
| 932 | P36897 | TGFR1_HUMAN |
| 933 | P36941 | TNR3_HUMAN |
| 934 | P36955 | PEDF_HUMAN |
| 935 | P36959 | GMPR1_HUMAN |
| 936 | P36980 | FHR2_HUMAN |

| | Gene | Protein |
|---|---|---|
| 937 | P37023 | ACVL1_HUMAN |
| 938 | P37108 | SRP14_HUMAN |
| 939 | P37173 | TGFR2_HUMAN |
| 940 | P37235 | HPCL1_HUMAN |
| 941 | P37837 | TALDO_HUMAN |
| 942 | P37840 | SYUA_HUMAN |
| 943 | P38484 | INGR2_HUMAN |
| 944 | P38935 | SMBP2_HUMAN |
| 945 | P38936 | CDN1A_HUMAN |
| 946 | P39059 | COFA1_HUMAN |
| 947 | P39060 | COIA1_HUMAN |
| 948 | P39748 | FEN1_HUMAN |
| 949 | P39900 | MMP12_HUMAN |
| 950 | P39905 | GDNF_HUMAN |
| 951 | P40121 | CAPG_HUMAN |
| 952 | P40189 | IL6RB_HUMAN |
| 953 | P40197 | GPV_HUMAN |
| 954 | P40199 | CEAM6_HUMAN |
| 955 | P40222 | TXLNA_HUMAN |
| 956 | P40225 | TPO_HUMAN |
| 957 | P40259 | CD79B_HUMAN |
| 958 | P40313 | CTRL_HUMAN |
| 959 | P40818 | UBP8_HUMAN |
| 960 | P40933 | IL15_HUMAN |
| 961 | P41159 | LEP_HUMAN |
| 962 | P41208 | CETN2_HUMAN |
| 963 | P41217 | OX2G_HUMAN |
| 964 | P41218 | MNDA_HUMAN |
| 965 | P41222 | PTGDS_HUMAN |
| 966 | P41227 | NAA10_HUMAN |
| 967 | P41236 | IPP2_HUMAN |
| 968 | P41271 | NBL1_HUMAN |
| 969 | P41273 | TNFL9_HUMAN |
| 970 | P41439 | FOLR3_HUMAN |
| 971 | P41586 | PACR_HUMAN |
| 972 | P42081 | CD86_HUMAN |
| 973 | P42331 | RHG25_HUMAN |
| 974 | P42574 | CASP3_HUMAN |
| 975 | P42575 | CASP2_HUMAN |
| 976 | P42658 | DPP6_HUMAN |
| 977 | P42701 | I12R1_HUMAN |
| 978 | P42702 | LIFR_HUMAN |
| 979 | P42785 | PCP_HUMAN |
| 980 | P42830 | CXCL5_HUMAN |
| 981 | P42892 | ECE1_HUMAN |
| 982 | P43121 | MUC18_HUMAN |
| 983 | P43234 | CATO_HUMAN |
| 984 | P43251 | BTD_HUMAN |
| 985 | P43320 | CRBB2_HUMAN |
| 986 | P43487 | RANG_HUMAN |
| 987 | P43489 | TNR4_HUMAN |
| 988 | P43490 | NAMPT_HUMAN |
| 989 | P43627 | KI2L2_HUMAN |
| 990 | P43628 | KI2L3_HUMAN |
| 991 | P43629 | KI3L1_HUMAN |
| 992 | P43630 | KI3L2_HUMAN |
| 993 | P43632 | KI2S4_HUMAN |
| 994 | P43652 | AFAM_HUMAN |
| 995 | P45452 | MMP13_HUMAN |
| 996 | P45954 | ACDSB_HUMAN |
| 997 | P45984 | MK09_HUMAN |
| 998 | P46013 | KI67_HUMAN |
| 999 | P46109 | CRKL_HUMAN |
| 1000 | P46379 | BAG6_HUMAN |
| 1001 | P46531 | NOTC1_HUMAN |
| 1002 | P46926 | GNPI1_HUMAN |
| 1003 | P47712 | PA24A_HUMAN |
| 1004 | P47929 | LEG7_HUMAN |
| 1005 | P47972 | NPTX2_HUMAN |
| 1006 | P47992 | XCL1_HUMAN |
| 1007 | P48023 | TNFL6_HUMAN |
| 1008 | P48052 | CBPA2_HUMAN |
| 1009 | P48060 | GLIP1_HUMAN |
| 1010 | P48061 | SDF1_HUMAN |
| 1011 | P48304 | REG1B_HUMAN |
| 1012 | P48307 | TFPI2_HUMAN |
| 1013 | P48357 | LEPR_HUMAN |
| 1014 | P48507 | GSH0_HUMAN |
| 1015 | P48740 | MASP1_HUMAN |
| 1016 | P48745 | CCN3_HUMAN |
| 1017 | P48775 | T23O_HUMAN |
| 1018 | P48960 | AGRE5_HUMAN |
| 1019 | P49023 | PAXI_HUMAN |
| 1020 | P49069 | CAMLG_HUMAN |
| 1021 | P49137 | MAPK2_HUMAN |
| 1022 | P49223 | SPIT3_HUMAN |
| 1023 | P49354 | FNTA_HUMAN |
| 1024 | P49366 | DHYS_HUMAN |
| 1025 | P49441 | INPP_HUMAN |
| 1026 | P49593 | PPM1F_HUMAN |
| 1027 | P49662 | CASP4_HUMAN |
| 1028 | P49715 | CEBPA_HUMAN |
| 1029 | P49747 | COMP_HUMAN |
| 1030 | P49757 | NUMB_HUMAN |
| 1031 | P49763 | PLGF_HUMAN |
| 1032 | P49765 | VEGFB_HUMAN |
| 1033 | P49767 | VEGFC_HUMAN |
| 1034 | P49771 | FLT3L_HUMAN |
| 1035 | P49788 | TIG1_HUMAN |
| 1036 | P49789 | FHIT_HUMAN |
| 1037 | P49862 | KLK7_HUMAN |
| 1038 | P49908 | SEPP1_HUMAN |
| 1039 | P50053 | KHK_HUMAN |
| 1040 | P50120 | RET2_HUMAN |
| 1041 | P50135 | HNMT_HUMAN |
| 1042 | P50225 | ST1A1_HUMAN |
| 1043 | P50452 | SPB8_HUMAN |
| 1044 | P50453 | SPB9_HUMAN |
| 1045 | P50461 | CSRP3_HUMAN |
| 1046 | P50502 | F10A1_HUMAN |
| 1047 | P50552 | VASP_HUMAN |
| 1048 | P50579 | MAP2_HUMAN |
| 1049 | P50591 | TNF10_HUMAN |
| 1050 | P50749 | RASF2_HUMAN |
| 1051 | P50895 | BCAM_HUMAN |
| 1052 | P50897 | PPT1_HUMAN |
| 1053 | P50995 | ANX11_HUMAN |
| 1054 | P51161 | FABP6_HUMAN |
| 1055 | P51452 | DUS3_HUMAN |
| 1056 | P51580 | TPMT_HUMAN |
| 1057 | P51617 | IRAK1_HUMAN |
| 1058 | P51649 | SSDH_HUMAN |
| 1059 | P51671 | CCL11_HUMAN |
| 1060 | P51687 | SUOX_HUMAN |
| 1061 | P51688 | SPHM_HUMAN |
| 1062 | P51692 | STA5B_HUMAN |
| 1063 | P51693 | APLP1_HUMAN |
| 1064 | P51808 | DYLT3_HUMAN |
| 1065 | P51858 | HDGF_HUMAN |
| 1066 | P51888 | PRELP_HUMAN |
| 1067 | P52179 | MYOM1_HUMAN |
| 1068 | P52209 | 6PGD_HUMAN |
| 1069 | P52564 | MP2K6_HUMAN |
| 1070 | P52630 | STAT2_HUMAN |
| 1071 | P52758 | RIDA_HUMAN |
| 1072 | P52798 | EFNA4_HUMAN |
| 1073 | P52799 | EFNB2_HUMAN |
| 1074 | P52823 | STC1_HUMAN |
| 1075 | P52848 | NDST1_HUMAN |
| 1076 | P52888 | THOP1_HUMAN |
| 1077 | P52943 | CRIP2_HUMAN |
| 1078 | P53367 | ARFP1_HUMAN |
| 1079 | P53634 | CATC_HUMAN |
| 1080 | P53674 | CRBB1_HUMAN |
| 1081 | P53814 | SMTN_HUMAN |
| 1082 | P53985 | MOT1_HUMAN |
| 1083 | P53990 | IST1_HUMAN |
| 1084 | P54105 | ICLN_HUMAN |
| 1085 | P54108 | CRIS3_HUMAN |
| 1086 | P54252 | ATX3_HUMAN |
| 1087 | P54274 | TERF1_HUMAN |
| 1088 | P54277 | PMS1_HUMAN |
| 1089 | P54284 | CACB3_HUMAN |
| 1090 | P54315 | LIPR1_HUMAN |
| 1091 | P54317 | LIPR2_HUMAN |
| 1092 | P54577 | SYYC_HUMAN |
| 1093 | P98160 | PGBM_HUMAN |
| 1094 | P98161 | PKD1_HUMAN |
| 1095 | P98164 | LRP2_HUMAN |
| 1096 | P98170 | XIAP_HUMAN |
| 1097 | Q00722 | PLCB2_HUMAN |
| 1098 | Q00796 | DHSO_HUMAN |
| 1099 | Q00872 | MYPC1_HUMAN |
| 1100 | Q01151 | CD83_HUMAN |
| 1101 | Q01344 | IL5RA_HUMAN |
| 1102 | Q01432 | AMPD3_HUMAN |
| 1103 | Q01459 | DIAC_HUMAN |
| 1104 | Q01469 | FABP5_HUMAN |
| 1105 | Q01484 | ANK2_HUMAN |
| 1106 | Q01638 | ILRL1_HUMAN |
| 1107 | Q01826 | SATB1_HUMAN |
| 1108 | Q01973 | ROR1_HUMAN |
| 1109 | Q02083 | NAAA_HUMAN |
| 1110 | Q02223 | TNR17_HUMAN |
| 1111 | Q02246 | CNTN2_HUMAN |
| 1112 | Q02487 | DSC2_HUMAN |
| 1113 | Q02742 | GCNT1_HUMAN |
| 1114 | Q02747 | GUC2A_HUMAN |
| 1115 | Q02750 | MP2K1_HUMAN |
| 1116 | Q02763 | TIE2_HUMAN |
| 1117 | Q02790 | FKBP4_HUMAN |
| 1118 | Q02817 | MUC2_HUMAN |
| 1119 | Q02880 | TOP2B_HUMAN |
| 1120 | Q02952 | AKA12_HUMAN |
| 1121 | Q03014 | HHEX_HUMAN |
| 1122 | Q03154 | ACY1_HUMAN |
| 1123 | Q03167 | TGBR3_HUMAN |
| 1124 | Q03169 | TNAP2_HUMAN |
| 1125 | Q03252 | LMNB2_HUMAN |
| 1126 | Q03393 | PTPS_HUMAN |
| 1127 | Q03403 | TFF2_HUMAN |
| 1128 | Q03405 | UPAR_HUMAN |
| 1129 | Q03426 | KIME_HUMAN |
| 1130 | Q03431 | PTH1R_HUMAN |
| 1131 | Q04323 | UBXN1_HUMAN |
| 1132 | Q04609 | FOLH1_HUMAN |
| 1133 | Q04637 | IF4G1_HUMAN |
| 1134 | Q04721 | NOTC2_HUMAN |
| 1135 | Q04756 | HGFA_HUMAN |
| 1136 | Q04760 | LGUL_HUMAN |
| 1137 | Q04837 | SSBP_HUMAN |
| 1138 | Q04900 | MUC24_HUMAN |
| 1139 | Q05084 | ICA69_HUMAN |
| 1140 | Q05193 | DYN1_HUMAN |
| 1141 | Q05315 | LEG10_HUMAN |
| 1142 | Q05516 | ZBT16_HUMAN |
| 1143 | Q06033 | ITIH3_HUMAN |
| 1144 | Q06141 | REG3A_HUMAN |
| 1145 | Q06323 | PSME1_HUMAN |
| 1146 | Q06418 | TYRO3_HUMAN |
| 1147 | Q06520 | ST2A1_HUMAN |
| 1148 | Q06609 | RAD51_HUMAN |
| 1149 | Q06643 | TNFC_HUMAN |
| 1150 | Q06830 | PRDX1_HUMAN |
| 1151 | Q07011 | TNR9_HUMAN |
| 1152 | Q07065 | CKAP4_HUMAN |
| 1153 | Q07108 | CD69_HUMAN |
| 1154 | Q07325 | CXCL9_HUMAN |
| 1155 | Q07507 | DERM_HUMAN |
| 1156 | Q07617 | SPAG1_HUMAN |
| 1157 | Q07812 | BAX_HUMAN |
| 1158 | Q07817 | B2CL1_HUMAN |
| 1159 | Q07954 | LRP1_HUMAN |
| 1160 | Q07960 | RHG01_HUMAN |
| 1161 | Q08174 | PCDH1_HUMAN |
| 1162 | Q08334 | I10R2_HUMAN |
| 1163 | Q08345 | DDR1_HUMAN |
| 1164 | Q08378 | GOGA3_HUMAN |
| 1165 | Q08380 | LG3BP_HUMAN |
| 1166 | Q08431 | MFGM_HUMAN |
| 1167 | Q08629 | TICN1_HUMAN |
| 1168 | Q08708 | CLM6_HUMAN |
| 1169 | Q08830 | FGL1_HUMAN |
| 1170 | Q08AG7 | MZT1_HUMAN |
| 1171 | Q09666 | AHNK_HUMAN |
| 1172 | Q0VD83 | APOBR_HUMAN |
| 1173 | Q0Z7S8 | FABP9_HUMAN |
| 1174 | Q10471 | GALT2_HUMAN |
| 1175 | Q10588 | BST1_HUMAN |
| 1176 | Q10589 | BST2_HUMAN |
| 1177 | Q11201 | SIA4A_HUMAN |
| 1178 | Q12765 | SCRN1_HUMAN |
| 1179 | Q12774 | ARHG5_HUMAN |
| 1180 | Q12778 | FOXO1_HUMAN |
| 1181 | Q12794 | HYAL1_HUMAN |
| 1182 | Q12805 | FBLN3_HUMAN |
| 1183 | Q12841 | FSTL1_HUMAN |
| 1184 | Q12846 | STX4_HUMAN |
| 1185 | Q12860 | CNTN1_HUMAN |
| 1186 | Q12864 | CAD17_HUMAN |
| 1187 | Q12866 | MERTK_HUMAN |
| 1188 | Q12884 | SEPR_HUMAN |
| 1189 | Q12888 | TP53B_HUMAN |
| 1190 | Q12912 | IRAG2_HUMAN |
| 1191 | Q12918 | KLRB1_HUMAN |
| 1192 | Q12933 | TRAF2_HUMAN |
| 1193 | Q13002 | GRIK2_HUMAN |
| 1194 | Q13017 | RHG05_HUMAN |
| 1195 | Q13093 | PAFA_HUMAN |
| 1196 | Q13105 | ZBT17_HUMAN |
| 1197 | Q13114 | TRAF3_HUMAN |
| 1198 | Q13137 | CACO2_HUMAN |
| 1199 | Q13158 | FADD_HUMAN |
| 1200 | Q13219 | PAPP1_HUMAN |
| 1201 | Q13224 | NMDE2_HUMAN |
| 1202 | Q13231 | CHIT1_HUMAN |
| 1203 | Q13232 | NDK3_HUMAN |
| 1204 | Q13241 | KLRD1_HUMAN |
| 1205 | Q13261 | I15RA_HUMAN |
| 1206 | Q13275 | SEM3F_HUMAN |
| 1207 | Q13277 | STX3_HUMAN |
| 1208 | Q13291 | SLAF1_HUMAN |
| 1209 | Q13308 | PTK7_HUMAN |
| 1210 | Q13316 | DMP1_HUMAN |
| 1211 | Q13332 | PTPRS_HUMAN |
| 1212 | Q13361 | MFAP5_HUMAN |
| 1213 | Q13410 | BT1A1_HUMAN |
| 1214 | Q13421 | MSLN_HUMAN |
| 1215 | Q13428 | TCOF_HUMAN |
| 1216 | Q13442 | HAP28_HUMAN |
| 1217 | Q13443 | ADAM9_HUMAN |
| 1218 | Q13444 | ADA15_HUMAN |
| 1219 | Q13445 | TMED1_HUMAN |
| 1220 | Q13451 | FKBP5_HUMAN |
| 1221 | Q13459 | MYO9B_HUMAN |
| 1222 | Q13478 | IL18R_HUMAN |
| 1223 | Q13508 | NAR3_HUMAN |
| 1224 | Q13510 | ASAH1_HUMAN |
| 1225 | Q13541 | 4EBP1_HUMAN |
| 1226 | Q13561 | DCTN2_HUMAN |
| 1227 | Q13563 | PKD2_HUMAN |
| 1228 | Q13576 | IQGA2_HUMAN |
| 1229 | Q13651 | I10R1_HUMAN |
| 1230 | Q13740 | CD166_HUMAN |
| 1231 | Q13790 | APOF_HUMAN |
| 1232 | Q13822 | ENPP2_HUMAN |
| 1233 | Q13867 | BLMH_HUMAN |
| 1234 | Q13938 | CAYP1_HUMAN |
| 1235 | Q13976 | KGP1_HUMAN |
| 1236 | Q14005 | IL16_HUMAN |
| 1237 | Q14011 | CIRBP_HUMAN |
| 1238 | Q14088 | RB33A_HUMAN |
| 1239 | Q14108 | SCRB2_HUMAN |
| 1240 | Q14112 | NID2_HUMAN |
| 1241 | Q14116 | IL18_HUMAN |
| 1242 | Q14118 | DAG1_HUMAN |
| 1243 | Q14126 | DSG2_HUMAN |
| 1244 | Q14129 | DGCR6_HUMAN |
| 1245 | Q14149 | MORC3_HUMAN |
| 1246 | Q14151 | SAFB2_HUMAN |
| 1247 | Q14160 | SCRIB_HUMAN |
| 1248 | Q14162 | SREC_HUMAN |

| | Gene | Protein | | Gene | Protein | | Gene | Protein | | Gene | Protein |
|---|---|---|---|---|---|---|---|---|---|---|---|
| 1249 | Q14184 | DOC2B_HUMAN | 1327 | P54652 | HSP72_HUMAN | 1405 | Q16627 | CCL14_HUMAN | 1483 | Q6BCY4 | NB5R2_HUMAN |
| 1250 | Q14203 | DCTN1_HUMAN | 1328 | P54687 | BCAT1_HUMAN | 1406 | Q16643 | DREB_HUMAN | 1484 | Q6DN72 | FCRL6_HUMAN |
| 1251 | Q14210 | LY6D_HUMAN | 1329 | P54727 | RD23B_HUMAN | 1407 | Q16651 | PRSS8_HUMAN | 1485 | Q6EIG7 | CLC6A_HUMAN |
| 1252 | Q14241 | ELOA1_HUMAN | 1330 | P54760 | EPHB4_HUMAN | 1408 | Q16653 | MOG_HUMAN | 1486 | Q6EMK4 | VASN_HUMAN |
| 1253 | Q14242 | SELPL_HUMAN | 1331 | P54764 | EPHA4_HUMAN | 1409 | Q16663 | CCL15_HUMAN | 1487 | Q6FHJ7 | SFRP4_HUMAN |
| 1254 | Q14246 | AGRE1_HUMAN | 1332 | P54819 | KAD2_HUMAN | 1410 | Q16674 | MIA_HUMAN | 1488 | Q6FI81 | CPIN1_HUMAN |
| 1255 | Q14258 | TRI25_HUMAN | 1333 | P55000 | SLUR1_HUMAN | 1411 | Q16698 | DECR_HUMAN | 1489 | Q6GMV3 | PTRD1_HUMAN |
| 1256 | Q14324 | MYPC2_HUMAN | 1334 | P55058 | PLTP_HUMAN | 1412 | Q16719 | KYNU_HUMAN | 1490 | Q6GQQ9 | OTU7B_HUMAN |
| 1257 | Q14393 | GAS6_HUMAN | 1335 | P55082 | MFAP3_HUMAN | 1413 | Q16740 | CLPP_HUMAN | 1491 | Q6GTS8 | P20D1_HUMAN |
| 1258 | Q14435 | GALT3_HUMAN | 1336 | P55083 | MFAP4_HUMAN | 1414 | Q16769 | QPCT_HUMAN | 1492 | Q6GTX8 | LAIR1_HUMAN |
| 1259 | Q14457 | BECN1_HUMAN | 1337 | P55103 | INHBC_HUMAN | 1415 | Q16772 | GSTA3_HUMAN | 1493 | Q6IBS0 | TWF2_HUMAN |
| 1260 | Q14508 | WFDC2_HUMAN | 1338 | P55145 | MANF_HUMAN | 1416 | Q16773 | KAT1_HUMAN | 1494 | Q6ISS4 | LAIR2_HUMAN |
| 1261 | Q14511 | CASL_HUMAN | 1339 | P55210 | CASP7_HUMAN | 1417 | Q16775 | GLO2_HUMAN | 1495 | Q6N021 | TET2_HUMAN |
| 1262 | Q14512 | FGFP1_HUMAN | 1340 | P55211 | CASP9_HUMAN | 1418 | Q16790 | CAH9_HUMAN | 1496 | Q6NUJ1 | SAPL1_HUMAN |
| 1263 | Q14515 | SPRL1_HUMAN | 1341 | P55259 | GP2_HUMAN | 1419 | Q16819 | MEP1A_HUMAN | 1497 | Q6NUS6 | TECT3_HUMAN |
| 1264 | Q14554 | PDIA5_HUMAN | 1342 | P55273 | CDN2D_HUMAN | 1420 | Q16820 | MEP1B_HUMAN | 1498 | Q6NW40 | RGMB_HUMAN |
| 1265 | Q14624 | ITIH4_HUMAN | 1343 | P55285 | CADH6_HUMAN | 1421 | Q16853 | AOC3_HUMAN | 1499 | Q6NXT1 | ANR54_HUMAN |
| 1266 | Q14641 | INSL4_HUMAN | 1344 | P55291 | CAD15_HUMAN | 1422 | Q16864 | VATF_HUMAN | 1500 | Q6P1J6 | PLB1_HUMAN |
| 1267 | Q14643 | ITPR1_HUMAN | 1345 | P55773 | CCL23_HUMAN | 1423 | Q16881 | TRXR1_HUMAN | 1501 | Q6P1M0 | S27A4_HUMAN |
| 1268 | Q14696 | MESD_HUMAN | 1346 | P55774 | CCL18_HUMAN | 1424 | Q16891 | MIC60_HUMAN | 1502 | Q6P1N0 | C2D1A_HUMAN |
| 1269 | Q14739 | LBR_HUMAN | 1347 | P55789 | ALR_HUMAN | 1425 | Q17R60 | IMPG1_HUMAN | 1503 | Q6P2H3 | CEP85_HUMAN |
| 1270 | Q14767 | LTBP2_HUMAN | 1348 | P55808 | XG_HUMAN | 1426 | Q29980 | MICB_HUMAN | 1504 | Q6P4E1 | GOLM2_HUMAN |
| 1271 | Q14773 | ICAM4_HUMAN | 1349 | P55809 | SCOT1_HUMAN | 1427 | Q2L4Q9 | PRS53_HUMAN | 1505 | Q6P589 | TP8L2_HUMAN |
| 1272 | Q14790 | CASP8_HUMAN | 1350 | P56159 | GFRA1_HUMAN | 1428 | Q2MKA7 | RSPO1_HUMAN | 1506 | Q6P5Z2 | PKN3_HUMAN |
| 1273 | Q14894 | CRYM_HUMAN | 1351 | P56192 | SYMC_HUMAN | 1429 | Q2TAL6 | VWC2_HUMAN | 1507 | Q6P995 | F171B_HUMAN |
| 1274 | Q14914 | PTGR1_HUMAN | 1352 | P56279 | TCL1A_HUMAN | 1430 | Q2UY09 | COSA1_HUMAN | 1508 | Q6PCB0 | VWA1_HUMAN |
| 1275 | Q14956 | GPNMB_HUMAN | 1353 | P56470 | LEG4_HUMAN | 1431 | Q2VWP7 | PRTG_HUMAN | 1509 | Q6PGN9 | PSRC1_HUMAN |
| 1276 | Q14BN4 | SLMAP_HUMAN | 1354 | P57087 | JAM2_HUMAN | 1432 | Q2WEN9 | CEA16_HUMAN | 1510 | Q6PI73 | LIRA6_HUMAN |
| 1277 | Q15018 | ABRX2_HUMAN | 1355 | P58107 | EPIPL_HUMAN | 1433 | Q32MZ4 | LRRF1_HUMAN | 1511 | Q6PJW8 | CNST_HUMAN |
| 1278 | Q15025 | TNIP1_HUMAN | 1356 | P58294 | PROK1_HUMAN | 1434 | Q3KPI0 | CEA21_HUMAN | 1512 | Q6PKG0 | LARP1_HUMAN |
| 1279 | Q15043 | S39AE_HUMAN | 1357 | P58499 | FAM3B_HUMAN | 1435 | Q495A1 | TIGIT_HUMAN | 1513 | Q6PKH6 | DR4L2_HUMAN |
| 1280 | Q15063 | POSTN_HUMAN | 1358 | P59665 | DEF1_HUMAN | 1436 | Q496F6 | CLM2_HUMAN | 1514 | Q6PL24 | TMED8_HUMAN |
| 1281 | Q15109 | RAGE_HUMAN | 1359 | P59780 | AP3S2_HUMAN | 1437 | Q49A26 | GLYR1_HUMAN | 1515 | Q6QNK2 | AGRD1_HUMAN |
| 1282 | Q15113 | PCOC1_HUMAN | 1360 | P60880 | SNP25_HUMAN | 1438 | Q49AH0 | CDNF_HUMAN | 1516 | Q6QNY0 | BL1S3_HUMAN |
| 1283 | Q15116 | PDCD1_HUMAN | 1361 | P61026 | RAB10_HUMAN | 1439 | Q4KMG0 | CDON_HUMAN | 1517 | Q6UB28 | MAP12_HUMAN |
| 1284 | Q15126 | PMVK_HUMAN | 1362 | P61218 | RPAB2_HUMAN | 1440 | Q4VCS5 | AMOT_HUMAN | 1518 | Q6UVK1 | CSPG4_HUMAN |
| 1285 | Q15155 | NOMO1_HUMAN | 1363 | P61266 | STX1B_HUMAN | 1441 | Q4ZHG4 | FNDC1_HUMAN | 1519 | Q6UW15 | REG3G_HUMAN |
| 1286 | Q15165 | PON2_HUMAN | 1364 | P61366 | OSTN_HUMAN | 1442 | Q53EL9 | SEZ6_HUMAN | 1520 | Q6UW49 | SPESP_HUMAN |
| 1287 | Q15166 | PON3_HUMAN | 1365 | P61457 | PHS_HUMAN | 1443 | Q53FA7 | QORX_HUMAN | 1521 | Q6UW56 | ARAID_HUMAN |
| 1288 | Q15172 | 2A5A_HUMAN | 1366 | P61769 | B2MG_HUMAN | 1444 | Q53GL0 | PKHO1_HUMAN | 1522 | Q6UWK7 | GP15L_HUMAN |
| 1289 | Q15223 | NECT1_HUMAN | 1367 | P61812 | TGFB2_HUMAN | 1445 | Q53H47 | SETMR_HUMAN | 1523 | Q6UWL2 | SUSD1_HUMAN |
| 1290 | Q15256 | PTPRR_HUMAN | 1368 | P61916 | NPC2_HUMAN | 1446 | Q53H82 | LACB2_HUMAN | 1524 | Q6UWL6 | KIRR2_HUMAN |
| 1291 | Q15262 | PTPRK_HUMAN | 1369 | P61978 | HNRPK_HUMAN | 1447 | Q53T59 | H1BP3_HUMAN | 1525 | Q6UWN8 | ISK6_HUMAN |
| 1292 | Q15276 | RABE1_HUMAN | 1370 | P62166 | NCS1_HUMAN | 1448 | Q58EX2 | SDK2_HUMAN | 1526 | Q6UWP8 | SBSN_HUMAN |
| 1293 | Q15303 | ERBB4_HUMAN | 1371 | P62330 | ARF6_HUMAN | 1449 | Q5FWE3 | PRRT3_HUMAN | 1527 | Q6UWR7 | ENPP6_HUMAN |
| 1294 | Q15366 | PCBP2_HUMAN | 1372 | P62736 | ACTA_HUMAN | 1450 | Q5GAN6 | RNS10_HUMAN | 1528 | Q6UWV6 | ENPP7_HUMAN |
| 1295 | Q15388 | TOM20_HUMAN | 1373 | P62760 | VISL1_HUMAN | 1451 | Q5JS37 | NHLC3_HUMAN | 1529 | Q6UWW0 | LCN15_HUMAN |
| 1296 | Q15389 | ANGP1_HUMAN | 1374 | P63098 | CANB1_HUMAN | 1452 | Q5JS54 | PSMG4_HUMAN | 1530 | Q6UWW8 | EST3_HUMAN |
| 1297 | Q15398 | DLGP5_HUMAN | 1375 | P63146 | UBE2B_HUMAN | 1453 | Q5JSP0 | FGD3_HUMAN | 1531 | Q6UX06 | OLFM4_HUMAN |
| 1298 | Q15399 | TLR1_HUMAN | 1376 | P63172 | DYLT1_HUMAN | 1454 | Q5JTD0 | TJAP1_HUMAN | 1532 | Q6UX15 | LAYN_HUMAN |
| 1299 | Q15427 | SF3B4_HUMAN | 1377 | P63313 | TYB10_HUMAN | 1455 | Q5JTV8 | TOIP1_HUMAN | 1533 | Q6UX27 | VSTM1_HUMAN |
| 1300 | Q15485 | FCN2_HUMAN | 1378 | P68106 | FKB1B_HUMAN | 1456 | Q5KU26 | COL12_HUMAN | 1534 | Q6UX71 | PXDC2_HUMAN |
| 1301 | Q15517 | CDSN_HUMAN | 1379 | P78310 | CXAR_HUMAN | 1457 | Q5QGZ9 | CL12A_HUMAN | 1535 | Q6UX82 | LYPD8_HUMAN |
| 1302 | Q15582 | BGH3_HUMAN | 1380 | P78318 | IGBP1_HUMAN | 1458 | Q5R372 | RBG1L_HUMAN | 1536 | Q6UXB2 | CXL17_HUMAN |
| 1303 | Q15599 | NHRF2_HUMAN | 1381 | P78324 | SHPS1_HUMAN | 1459 | Q5SW79 | CE170_HUMAN | 1537 | Q6UXB4 | CLC4G_HUMAN |
| 1304 | Q15633 | TRBP2_HUMAN | 1382 | P78325 | ADAM8_HUMAN | 1460 | Q5T2D2 | TRML2_HUMAN | 1538 | Q6UXB8 | PI16_HUMAN |
| 1305 | Q15661 | TRYB1_HUMAN | 1383 | P78333 | GPC5_HUMAN | 1461 | Q5T2W1 | NHRF3_HUMAN | 1539 | Q6UXC1 | AEGP_HUMAN |
| 1306 | Q15735 | PI5PA_HUMAN | 1384 | P78352 | DLG4_HUMAN | 1462 | Q5T5Y3 | CAMP1_HUMAN | 1540 | Q6UXD5 | SE6L2_HUMAN |
| 1307 | Q15762 | CD226_HUMAN | 1385 | P78362 | SRPK2_HUMAN | 1463 | Q5T848 | MGLYR_HUMAN | 1541 | Q6UXG3 | CLM9_HUMAN |
| 1308 | Q15796 | SMAD2_HUMAN | 1386 | P78380 | OLR1_HUMAN | 1464 | Q5TBC7 | B2L15_HUMAN | 1542 | Q6UXH1 | CREL2_HUMAN |
| 1309 | Q15814 | TBCC_HUMAN | 1387 | P78410 | BT3A2_HUMAN | 1465 | Q5TDH0 | DDI2_HUMAN | 1543 | Q6UXH9 | PAMR1_HUMAN |
| 1310 | Q15818 | NPTX1_HUMAN | 1388 | P78423 | X3CL1_HUMAN | 1466 | Q5VIR6 | VPS53_HUMAN | 1544 | Q6UXK2 | ISLR2_HUMAN |
| 1311 | Q15828 | CYTM_HUMAN | 1389 | P78539 | SRPX_HUMAN | 1467 | Q5VSG8 | MANEL_HUMAN | 1545 | Q6UXK5 | LRRN1_HUMAN |
| 1312 | Q15831 | STK11_HUMAN | 1390 | P78552 | I13R1_HUMAN | 1468 | Q5VT99 | LRC38_HUMAN | 1546 | Q6UXM1 | LRIG3_HUMAN |
| 1313 | Q15846 | CLUL1_HUMAN | 1391 | P78556 | CCL20_HUMAN | 1469 | Q5VTT5 | MYOM3_HUMAN | 1547 | Q6UXV0 | GFRAL_HUMAN |
| 1314 | Q16181 | SEPT7_HUMAN | 1392 | P78560 | CRADD_HUMAN | 1470 | Q5VV43 | K0319_HUMAN | 1548 | Q6UXZ4 | UNC5D_HUMAN |
| 1315 | Q16270 | IBP7_HUMAN | 1393 | P80075 | CCL8_HUMAN | 1471 | Q5VVQ6 | OTU1_HUMAN | 1549 | Q6UY14 | ATL4_HUMAN |
| 1316 | Q16288 | NTRK3_HUMAN | 1394 | P80098 | CCL7_HUMAN | 1472 | Q5VX71 | SUSD4_HUMAN | 1550 | Q6WCQ1 | MPRIP_HUMAN |
| 1317 | Q16363 | LAMA4_HUMAN | 1395 | P80162 | CXCL6_HUMAN | 1473 | Q5VY43 | PEAR1_HUMAN | 1551 | Q6WN34 | CRDL2_HUMAN |
| 1318 | Q16520 | BATF_HUMAN | 1396 | P80188 | NGAL_HUMAN | 1474 | Q5ZPR3 | CD276_HUMAN | 1552 | Q6XQN6 | PNCB_HUMAN |
| 1319 | Q16543 | CDC37_HUMAN | 1397 | P80303 | NUCB2_HUMAN | 1475 | Q63HQ2 | EGFLA_HUMAN | 1553 | Q6XZF7 | DNMBP_HUMAN |
| 1320 | Q16549 | PCSK7_HUMAN | 1398 | P80370 | DLK1_HUMAN | 1476 | Q674X7 | KAZRN_HUMAN | 1554 | Q6Y7W6 | GGYF2_HUMAN |
| 1321 | Q16552 | IL17_HUMAN | 1399 | P80511 | S10AC_HUMAN | 1477 | Q676U5 | A16L1_HUMAN | 1555 | Q6YHK3 | CD109_HUMAN |
| 1322 | Q16595 | FRDA_HUMAN | 1400 | P82980 | RET5_HUMAN | 1478 | Q68D85 | NR3L1_HUMAN | 1556 | Q6YN16 | HSDL2_HUMAN |
| 1323 | Q16610 | ECM1_HUMAN | 1401 | P85299 | PRR5_HUMAN | 1479 | Q68DV7 | RNF43_HUMAN | 1557 | Q6ZMC9 | SIG15_HUMAN |
| 1324 | Q16620 | NTRK2_HUMAN | 1402 | P98073 | ENTK_HUMAN | 1480 | Q68J44 | DUS29_HUMAN | 1558 | Q6ZMH5 | S39A5_HUMAN |
| 1325 | Q16621 | NFE2_HUMAN | 1403 | P98082 | DAB2_HUMAN | 1481 | Q6B8I1 | DS13A_HUMAN | 1559 | Q6ZMJ2 | SCAR5_HUMAN |
| 1326 | Q16625 | OCLN_HUMAN | 1404 | P98095 | FBLN2_HUMAN | 1482 | Q6BAA4 | FCRLB_HUMAN | 1560 | Q6ZMJ4 | IL34_HUMAN |

| | Gene | Protein |
|---|---|---|
| 1561 | Q6ZMM2 | ATL5_HUMAN |
| 1562 | Q6ZRY4 | RBPS2_HUMAN |
| 1563 | Q6ZUJ8 | BCAP_HUMAN |
| 1564 | Q6ZVN8 | RGMC_HUMAN |
| 1565 | Q765P7 | MTSS2_HUMAN |
| 1566 | Q76LX8 | ATS13_HUMAN |
| 1567 | Q76M96 | CCD80_HUMAN |
| 1568 | Q7KYR7 | BT2A1_HUMAN |
| 1569 | Q7L266 | ASGL1_HUMAN |
| 1570 | Q7L5N7 | PCAT2_HUMAN |
| 1571 | Q7L5Y9 | MAEA_HUMAN |
| 1572 | Q7LG56 | RIR2B_HUMAN |
| 1573 | Q7RTW8 | OTOAN_HUMAN |
| 1574 | Q7Z304 | MAMC2_HUMAN |
| 1575 | Q7Z3D4 | LYSM3_HUMAN |
| 1576 | Q7Z434 | MAVS_HUMAN |
| 1577 | Q7Z4V5 | HDGR2_HUMAN |
| 1578 | Q7Z4W1 | DCXR_HUMAN |
| 1579 | Q7Z4W2 | LYZL2_HUMAN |
| 1580 | Q7Z569 | BRAP_HUMAN |
| 1581 | Q7Z5A7 | TAFA5_HUMAN |
| 1582 | Q7Z5L0 | VMO1_HUMAN |
| 1583 | Q7Z5L3 | C1QL2_HUMAN |
| 1584 | Q7Z5R6 | AB1IP_HUMAN |
| 1585 | Q7Z692 | CEA19_HUMAN |
| 1586 | Q7Z6M3 | MILR1_HUMAN |
| 1587 | Q7Z6P3 | RAB44_HUMAN |
| 1588 | Q7Z7D3 | VTCN1_HUMAN |
| 1589 | Q7Z7M9 | GALT5_HUMAN |
| 1590 | Q86SF2 | GALT7_HUMAN |
| 1591 | Q86SJ2 | AMGO2_HUMAN |
| 1592 | Q86SJ6 | DSG4_HUMAN |
| 1593 | Q86SQ7 | SDCG8_HUMAN |
| 1594 | Q86SR1 | GLT10_HUMAN |
| 1595 | Q86SX6 | GLRX5_HUMAN |
| 1596 | Q86T13 | CLC14_HUMAN |
| 1597 | Q86TE4 | LUZP2_HUMAN |
| 1598 | Q86TH1 | ATL2_HUMAN |
| 1599 | Q86U17 | SPA11_HUMAN |
| 1600 | Q86UE4 | LYRIC_HUMAN |
| 1601 | Q86UU1 | PHLB1_HUMAN |
| 1602 | Q86UW2 | OSTB_HUMAN |
| 1603 | Q86UW9 | DTX2_HUMAN |
| 1604 | Q86VB7 | C163A_HUMAN |
| 1605 | Q86VP1 | TAXB1_HUMAN |
| 1606 | Q86VR7 | VS10L_HUMAN |
| 1607 | Q86VZ4 | LRP11_HUMAN |
| 1608 | Q86WD7 | SPA9_HUMAN |
| 1609 | Q86WV1 | SKAP1_HUMAN |
| 1610 | Q86X76 | NIT1_HUMAN |
| 1611 | Q86YD3 | TMM25_HUMAN |
| 1612 | Q86YW5 | TRML1_HUMAN |
| 1613 | Q86Z14 | KLOTB_HUMAN |
| 1614 | Q8IU54 | IFNL1_HUMAN |
| 1615 | Q8IU57 | INLR1_HUMAN |
| 1616 | Q8IUK5 | PLDX1_HUMAN |
| 1617 | Q8IUN9 | CLC10_HUMAN |
| 1618 | Q8IUZ5 | AT2L2_HUMAN |
| 1619 | Q8IV16 | HDBP1_HUMAN |
| 1620 | Q8IV38 | ANKY2_HUMAN |
| 1621 | Q8IVF2 | AHNK2_HUMAN |
| 1622 | Q8IVG5 | SAM9L_HUMAN |
| 1623 | Q8IVM0 | CCD50_HUMAN |
| 1624 | Q8IW75 | SPA12_HUMAN |
| 1625 | Q8IWB1 | IPRI_HUMAN |
| 1626 | Q8IWL1 | SFPA2_HUMAN |
| 1627 | Q8IWL2 | SFTA1_HUMAN |
| 1628 | Q8IWT1 | SCN4B_HUMAN |
| 1629 | Q8IWV2 | CNTN4_HUMAN |
| 1630 | Q8IWZ8 | SUGP1_HUMAN |
| 1631 | Q8IX05 | CD302_HUMAN |
| 1632 | Q8IX19 | MCEM1_HUMAN |
| 1633 | Q8IXJ6 | SIR2_HUMAN |
| 1634 | Q8IXM2 | BAP18_HUMAN |
| 1635 | Q8IXQ3 | CI040_HUMAN |
| 1636 | Q9Y2D5 | PLAK2_HUMAN |
| 1637 | Q8IY22 | CMIP_HUMAN |
| 1638 | Q8IY33 | MILK2_HUMAN |
| 1639 | Q8TF64 | GIPC3_HUMAN |
| 1640 | Q8TF65 | GIPC2_HUMAN |
| 1641 | Q8WTT0 | CLC4C_HUMAN |
| 1642 | Q8WTU2 | SRB4D_HUMAN |
| 1643 | Q8WTV0 | SCRB1_HUMAN |
| 1644 | Q8WU39 | MZB1_HUMAN |
| 1645 | Q8WUD1 | RAB2B_HUMAN |
| 1646 | Q8WUF8 | ARB2A_HUMAN |
| 1647 | Q8WUW1 | BRK1_HUMAN |
| 1648 | Q8WUX2 | CHAC2_HUMAN |
| 1649 | Q8WUY3 | PRUN2_HUMAN |
| 1650 | Q8WV28 | BLNK_HUMAN |
| 1651 | Q8WV92 | MITD1_HUMAN |
| 1652 | Q8WVC0 | LEO1_HUMAN |
| 1653 | Q8WVQ1 | CANT1_HUMAN |
| 1654 | Q8WVV4 | POF1B_HUMAN |
| 1655 | Q8WW22 | DNJA4_HUMAN |
| 1656 | Q8WWF5 | ZNRF4_HUMAN |
| 1657 | Q8WWN9 | ICEF1_HUMAN |
| 1658 | Q8WWQ8 | STAB2_HUMAN |
| 1659 | Q8WWV3 | RT4I1_HUMAN |
| 1660 | Q8WWV6 | FCAMR_HUMAN |
| 1661 | Q8WWY7 | WFD12_HUMAN |
| 1662 | Q8WX77 | IBPL1_HUMAN |
| 1663 | Q8WX93 | PALLD_HUMAN |
| 1664 | Q8WXC3 | PYDC1_HUMAN |
| 1665 | Q8WXD2 | SCG3_HUMAN |
| 1666 | Q8WXI7 | MUC16_HUMAN |
| 1667 | Q8WXI8 | CLC4D_HUMAN |
| 1668 | Q8WXW3 | PIBF1_HUMAN |
| 1669 | Q8WXX5 | DNJC9_HUMAN |
| 1670 | Q8WYN0 | ATG4A_HUMAN |
| 1671 | Q8WYQ3 | CHC10_HUMAN |
| 1672 | Q8WZ75 | ROBO4_HUMAN |
| 1673 | Q92185 | SIA8A_HUMAN |
| 1674 | Q92484 | ASM3A_HUMAN |
| 1675 | Q92485 | ASM3B_HUMAN |
| 1676 | Q92496 | FHR4_HUMAN |
| 1677 | Q92520 | FAM3C_HUMAN |
| 1678 | Q92574 | TSC1_HUMAN |
| 1679 | Q92583 | CCL17_HUMAN |
| 1680 | Q92597 | NDRG1_HUMAN |
| 1681 | Q92599 | SEPT8_HUMAN |
| 1682 | Q92609 | TBCD5_HUMAN |
| 1683 | Q92619 | HMHA1_HUMAN |
| 1684 | Q92686 | NEUG_HUMAN |
| 1685 | Q92692 | NECT2_HUMAN |
| 1686 | Q92752 | TENR_HUMAN |
| 1687 | Q92765 | SFRP3_HUMAN |
| 1688 | Q92783 | STAM1_HUMAN |
| 1689 | Q92820 | GGH_HUMAN |
| 1690 | Q92823 | NRCAM_HUMAN |
| 1691 | Q92832 | NELL1_HUMAN |
| 1692 | Q92835 | SHIP1_HUMAN |
| 1693 | Q92854 | SEM4D_HUMAN |
| 1694 | Q92859 | NEO1_HUMAN |
| 1695 | Q92876 | KLK6_HUMAN |
| 1696 | Q92888 | ARHG1_HUMAN |
| 1697 | Q92890 | UFD1_HUMAN |
| 1698 | Q92932 | PTPR2_HUMAN |
| 1699 | Q92956 | TNR14_HUMAN |
| 1700 | Q92982 | NINJ1_HUMAN |
| 1701 | Q93015 | NAA80_HUMAN |
| 1702 | Q93033 | IGSF2_HUMAN |
| 1703 | Q93091 | RNAS6_HUMAN |
| 1704 | Q969D9 | TSLP_HUMAN |
| 1705 | Q969H8 | MYDGF_HUMAN |
| 1706 | Q969P0 | IGSF8_HUMAN |
| 1707 | Q969X0 | RIPL2_HUMAN |
| 1708 | Q969Z4 | TR19L_HUMAN |
| 1709 | Q96A00 | PP14A_HUMAN |
| 1710 | Q96A25 | T106A_HUMAN |
| 1711 | Q96A32 | MYL11_HUMAN |
| 1712 | Q96AP7 | ESAM_HUMAN |
| 1713 | Q96AQ6 | PBIP1_HUMAN |
| 1714 | Q96AX2 | RAB37_HUMAN |
| 1715 | Q96B36 | AKTS1_HUMAN |
| 1716 | Q96B86 | RGMA_HUMAN |
| 1717 | Q96BQ1 | FAM3D_HUMAN |
| 1718 | Q96C92 | ENTR1_HUMAN |
| 1719 | Q96CD2 | COAC_HUMAN |
| 1720 | Q96CG8 | CTHR1_HUMAN |
| 1721 | Q96CN9 | GCC1_HUMAN |
| 1722 | Q96D42 | HAVR1_HUMAN |
| 1723 | Q96DC8 | ECHD3_HUMAN |
| 1724 | Q96DE0 | NUD16_HUMAN |
| 1725 | Q96DR5 | BPIA2_HUMAN |
| 1726 | Q96DU3 | SLAF6_HUMAN |
| 1727 | Q96EK5 | KBP_HUMAN |
| 1728 | Q96EM0 | T3HPD_HUMAN |
| 1729 | Q96EU7 | C1GLC_HUMAN |
| 1730 | Q96F10 | SAT2_HUMAN |
| 1731 | Q96F46 | I17RA_HUMAN |
| 1732 | Q96FE7 | P3IP1_HUMAN |
| 1733 | Q96FQ6 | S10AG_HUMAN |
| 1734 | Q96FZ7 | CHMP6_HUMAN |
| 1735 | Q96GP6 | SREC2_HUMAN |
| 1736 | Q96GW7 | PGCB_HUMAN |
| 1737 | Q96H15 | TIMD4_HUMAN |
| 1738 | Q96HC4 | PDLI5_HUMAN |
| 1739 | Q96HD1 | CREL1_HUMAN |
| 1740 | Q96HD9 | ACY3_HUMAN |
| 1741 | Q96I15 | SCLY_HUMAN |
| 1742 | Q96I82 | KAZD1_HUMAN |
| 1743 | Q96ID5 | IGS21_HUMAN |
| 1744 | Q96IQ7 | VSIG2_HUMAN |
| 1745 | Q96IU4 | ABHEB_HUMAN |
| 1746 | Q96IY4 | CBPB2_HUMAN |
| 1747 | Q96J42 | TXD15_HUMAN |
| 1748 | Q96J84 | KIRR1_HUMAN |
| 1749 | Q96JA1 | LRIG1_HUMAN |
| 1750 | Q96K21 | ANCHR_HUMAN |
| 1751 | Q96KG7 | MEG10_HUMAN |
| 1752 | Q96KJ4 | MSLNL_HUMAN |
| 1753 | Q96KN2 | CNDP1_HUMAN |
| 1754 | Q96LA5 | FCRL2_HUMAN |
| 1755 | Q96LA6 | FCRL1_HUMAN |
| 1756 | Q96LB8 | PGRP4_HUMAN |
| 1757 | Q96LC7 | SIG10_HUMAN |
| 1758 | Q96MK3 | FA20A_HUMAN |
| 1759 | Q96MM7 | H6ST2_HUMAN |
| 1760 | Q96NA2 | RILP_HUMAN |
| 1761 | Q96NB1 | CEP20_HUMAN |
| 1762 | Q96NY8 | NECT4_HUMAN |
| 1763 | Q96NZ8 | WFKN1_HUMAN |
| 1764 | Q96NZ9 | PRAP1_HUMAN |
| 1765 | Q96P31 | FCRL3_HUMAN |
| 1766 | Q96PD2 | DCBD2_HUMAN |
| 1767 | Q96PD4 | IL17F_HUMAN |
| 1768 | Q96PD5 | PGRP2_HUMAN |
| 1769 | Q96PL1 | SG3A2_HUMAN |
| 1770 | Q96PL5 | ERMAP_HUMAN |
| 1771 | Q96PQ0 | SORC2_HUMAN |
| 1772 | Q96PU5 | NED4L_HUMAN |
| 1773 | Q96PX8 | SLIK1_HUMAN |
| 1774 | Q96QH8 | LYZL5_HUMAN |
| 1775 | Q96QR1 | SG3A1_HUMAN |
| 1776 | Q96R05 | RET7_HUMAN |
| 1777 | Q96RD9 | FCRL5_HUMAN |
| 1778 | Q96RE7 | NACC1_HUMAN |
| 1779 | Q96RJ3 | TR13C_HUMAN |
| 1780 | Q96RT1 | ERBIN_HUMAN |
| 1781 | Q96RU2 | UBP28_HUMAN |
| 1782 | Q96SB3 | NEB2_HUMAN |
| 1783 | Q96SM3 | CPXM1_HUMAN |
| 1784 | Q96T91 | GPHA2_HUMAN |
| 1785 | Q99062 | CSF3R_HUMAN |
| 1786 | Q99075 | HBEGF_HUMAN |
| 1787 | Q99426 | TBCB_HUMAN |
| 1788 | Q99435 | NELL2_HUMAN |
| 1789 | Q99447 | PCY2_HUMAN |
| 1790 | Q99497 | PARK7_HUMAN |
| 1791 | Q99523 | SORT_HUMAN |
| 1792 | Q99536 | VAT1_HUMAN |
| 1793 | Q99538 | LGMN_HUMAN |
| 1794 | Q99549 | MPP8_HUMAN |
| 1795 | Q99574 | NEUS_HUMAN |
| 1796 | Q99584 | S10AD_HUMAN |
| 1797 | Q99616 | CCL13_HUMAN |
| 1798 | Q99650 | OSMR_HUMAN |
| 1799 | Q99674 | CGRE1_HUMAN |
| 1800 | Q99683 | M3K5_HUMAN |
| 1801 | Q99700 | ATX2_HUMAN |
| 1802 | Q99704 | DOK1_HUMAN |
| 1803 | Q99717 | SMAD5_HUMAN |
| 1804 | Q99727 | TIMP4_HUMAN |
| 1805 | Q99731 | CCL19_HUMAN |
| 1806 | Q99733 | NP1L4_HUMAN |
| 1807 | Q99795 | GPA33_HUMAN |
| 1808 | Q99807 | COQ7_HUMAN |
| 1809 | Q99895 | CTRC_HUMAN |
| 1810 | Q99942 | RNF5_HUMAN |
| 1811 | Q99969 | RARR2_HUMAN |
| 1812 | Q99972 | MYOC_HUMAN |
| 1813 | Q99983 | OMD_HUMAN |
| 1814 | Q99988 | GDF15_HUMAN |
| 1815 | Q9BPX1 | DHB14_HUMAN |
| 1816 | Q9BQ51 | PD1L2_HUMAN |
| 1817 | Q9BQB4 | SOST_HUMAN |
| 1818 | Q9BQR3 | PRS27_HUMAN |
| 1819 | Q9BQS7 | HEPH_HUMAN |
| 1820 | Q9BQT9 | CSTN3_HUMAN |
| 1821 | Q9BRF8 | CPPED_HUMAN |
| 1822 | Q9BRK3 | MXRA8_HUMAN |
| 1823 | Q9BRQ6 | MIC25_HUMAN |
| 1824 | Q9BS26 | ERP44_HUMAN |
| 1825 | Q9BS40 | LXN_HUMAN |
| 1826 | Q9BSG5 | RTBDN_HUMAN |
| 1827 | Q9BSL1 | UBAC1_HUMAN |
| 1828 | Q9BSW2 | EFC4B_HUMAN |
| 1829 | Q9BT73 | PSMG3_HUMAN |
| 1830 | Q9BTE6 | AASD1_HUMAN |
| 1831 | Q9BTK6 | PAGR1_HUMAN |
| 1832 | Q9BU02 | THTPA_HUMAN |
| 1833 | Q9BU40 | CRDL1_HUMAN |
| 1834 | Q9BUD6 | SPON2_HUMAN |
| 1835 | Q9BUE0 | MED18_HUMAN |
| 1836 | Q9BUH6 | PAXX_HUMAN |
| 1837 | Q9BUJ2 | HNRL1_HUMAN |
| 1838 | Q9BUN1 | MENT_HUMAN |
| 1839 | Q9BUP0 | EFHD1_HUMAN |
| 1840 | Q9BV20 | MTNA_HUMAN |
| 1841 | Q9BV40 | VAMP8_HUMAN |
| 1842 | Q9BV79 | MECR_HUMAN |
| 1843 | Q9BVM4 | GGACT_HUMAN |
| 1844 | Q9BW04 | SARG_HUMAN |
| 1845 | Q9BW30 | TPPP3_HUMAN |
| 1846 | Q9BWV1 | BOC_HUMAN |
| 1847 | Q9BX10 | GTPB2_HUMAN |
| 1848 | Q9BX67 | JAM3_HUMAN |
| 1849 | Q9BXD5 | NPL_HUMAN |
| 1850 | Q9BXI3 | 5NT1A_HUMAN |
| 1851 | Q9BXJ0 | C1QT5_HUMAN |
| 1852 | Q9BXJ1 | C1QT1_HUMAN |
| 1853 | Q9BXJ7 | AMNLS_HUMAN |
| 1854 | Q9BXN1 | ASPN_HUMAN |
| 1855 | Q9BXN2 | CLC7A_HUMAN |
| 1856 | Q9BXR6 | FHR5_HUMAN |
| 1857 | Q9BXY4 | RSPO3_HUMAN |
| 1858 | Q9BY14 | TX101_HUMAN |
| 1859 | Q9BY76 | ANGL4_HUMAN |
| 1860 | Q9BYC5 | FUT8_HUMAN |
| 1861 | Q9BYE9 | CDHR2_HUMAN |
| 1862 | Q9BYF1 | ACE2_HUMAN |
| 1863 | Q9BYH1 | SE6L1_HUMAN |
| 1864 | Q9BYJ0 | FGFP2_HUMAN |
| 1865 | Q9BYZ8 | REG4_HUMAN |
| 1866 | Q9BZC7 | ABCA2_HUMAN |
| 1867 | Q9BZE9 | ASPC1_HUMAN |
| 1868 | Q9BZJ3 | TRYD_HUMAN |
| 1869 | Q9BZM5 | ULBP2_HUMAN |
| 1870 | Q9BZR6 | RTN4R_HUMAN |
| 1871 | Q9BZW8 | CD244_HUMAN |
| 1872 | Q9BZZ2 | SN_HUMAN |

| | Gene | Protein |
|---|---|---|
| 1873 | Q8IYS2 | K2013_HUMAN |
| 1874 | Q8IYS5 | OSCAR_HUMAN |
| 1875 | Q8IZF2 | AGRF5_HUMAN |
| 1876 | Q8IZP9 | AGRG2_HUMAN |
| 1877 | Q8N0X7 | SPART_HUMAN |
| 1878 | Q8N0Z9 | VSI10_HUMAN |
| 1879 | Q8N111 | CEND_HUMAN |
| 1880 | Q8N114 | SHSA5_HUMAN |
| 1881 | Q8N129 | CNPY4_HUMAN |
| 1882 | Q8N149 | LIRA2_HUMAN |
| 1883 | Q8N163 | CCAR2_HUMAN |
| 1884 | Q8N1Q1 | CAH13_HUMAN |
| 1885 | Q8N386 | LRC25_HUMAN |
| 1886 | Q8N423 | LIRB2_HUMAN |
| 1887 | Q8N436 | CPXM2_HUMAN |
| 1888 | Q8N474 | SFRP1_HUMAN |
| 1889 | Q8N4C8 | MINK1_HUMAN |
| 1890 | Q8N4F0 | BPIB2_HUMAN |
| 1891 | Q8N5J2 | MINY1_HUMAN |
| 1892 | Q8N5S9 | KKCC1_HUMAN |
| 1893 | Q8N608 | DPP10_HUMAN |
| 1894 | Q8N668 | COMD1_HUMAN |
| 1895 | Q8N6C8 | LIRA3_HUMAN |
| 1896 | Q8N6M0 | OTU6B_HUMAN |
| 1897 | Q8N6P7 | I22R1_HUMAN |
| 1898 | Q8N6Q3 | CD177_HUMAN |
| 1899 | Q8N8R5 | CB069_HUMAN |
| 1900 | Q8N8S7 | ENAH_HUMAN |
| 1901 | Q8N8U9 | BMPER_HUMAN |
| 1902 | Q8N967 | LRTM2_HUMAN |
| 1903 | Q8N9I9 | DTX3_HUMAN |
| 1904 | Q8NBI3 | DRAXI_HUMAN |
| 1905 | Q8NBJ7 | SUMF2_HUMAN |
| 1906 | Q8NBK3 | SUMF1_HUMAN |
| 1907 | Q8NBP7 | PCSK9_HUMAN |
| 1908 | Q8NBS9 | TXND5_HUMAN |
| 1909 | Q8NBZ7 | UXS1_HUMAN |
| 1910 | Q8NC01 | CLC1A_HUMAN |
| 1911 | Q8NC42 | RN149_HUMAN |
| 1912 | Q8NCC3 | PAG15_HUMAN |
| 1913 | Q8ND71 | GIMA8_HUMAN |
| 1914 | Q8NDA2 | HMCN2_HUMAN |
| 1915 | Q8NDB2 | BANK1_HUMAN |
| 1916 | Q8NDI1 | EHBP1_HUMAN |
| 1917 | Q8NEB7 | ACRBP_HUMAN |
| 1918 | Q8NEU8 | DP13B_HUMAN |
| 1919 | Q8NEZ2 | VP37A_HUMAN |
| 1920 | Q8NFL0 | B3GN7_HUMAN |
| 1921 | Q8NFP4 | MDGA1_HUMAN |
| 1922 | Q8NFT8 | DNER_HUMAN |
| 1923 | Q8NG06 | TRI58_HUMAN |
| 1924 | Q8NHJ6 | LIRB4_HUMAN |
| 1925 | Q8NHL6 | LIRB1_HUMAN |
| 1926 | Q8NHP1 | ARK74_HUMAN |
| 1927 | Q8NHV1 | GIMA7_HUMAN |
| 1928 | Q8NHZ8 | CDC26_HUMAN |
| 1929 | Q8NI17 | IL31R_HUMAN |
| 1930 | Q8NI22 | MCFD2_HUMAN |
| 1931 | Q8TAD2 | IL17D_HUMAN |
| 1932 | Q8TAT2 | FGFP3_HUMAN |
| 1933 | Q8TBM8 | DJB14_HUMAN |
| 1934 | Q8TCD5 | NT5C_HUMAN |
| 1935 | Q8TCT1 | PHOP1_HUMAN |
| 1936 | Q8TCU4 | ALMS1_HUMAN |
| 1937 | Q8TCZ2 | C99L2_HUMAN |
| 1938 | Q8TD06 | AGR3_HUMAN |
| 1939 | Q8TD46 | MO2R1_HUMAN |
| 1940 | Q8TDL5 | BPIB1_HUMAN |
| 1941 | Q8TDQ0 | HAVR2_HUMAN |
| 1942 | Q8TDQ1 | CLM1_HUMAN |
| 1943 | Q8TDQ7 | GNPI2_HUMAN |
| 1944 | Q8TDX7 | NEK7_HUMAN |
| 1945 | Q8TDY8 | IGDC4_HUMAN |
| 1946 | Q8TE57 | ATS16_HUMAN |
| 1947 | Q8TE58 | ATS15_HUMAN |
| 1948 | Q8TEA8 | DTD1_HUMAN |
| 1949 | Q8TER0 | SNED1_HUMAN |
| 1950 | Q8TEU8 | WFKN2_HUMAN |
| 1951 | Q9C005 | DPY30_HUMAN |
| 1952 | Q9C035 | TRIM5_HUMAN |
| 1953 | Q9C0C4 | SEM4C_HUMAN |
| 1954 | Q9GZM7 | TINAL_HUMAN |
| 1955 | Q9GZN4 | BSSP4_HUMAN |
| 1956 | Q9GZT3 | SLIRP_HUMAN |
| 1957 | Q9GZT9 | EGLN1_HUMAN |
| 1958 | Q9GZV9 | FGF23_HUMAN |
| 1959 | Q9GZX6 | IL22_HUMAN |
| 1960 | Q9GZY6 | NTAL_HUMAN |
| 1961 | Q9GZZ8 | LACRT_HUMAN |
| 1962 | Q9H008 | LHPP_HUMAN |
| 1963 | Q9H0C8 | ILKAP_HUMAN |
| 1964 | Q9H0P0 | 5NT3A_HUMAN |
| 1965 | Q9H0U9 | TSYL1_HUMAN |
| 1966 | Q9H156 | SLIK2_HUMAN |
| 1967 | Q9H171 | ZBP1_HUMAN |
| 1968 | Q9H173 | SIL1_HUMAN |
| 1969 | Q9H1U4 | MEGF9_HUMAN |
| 1970 | Q9H251 | CAD23_HUMAN |
| 1971 | Q9H2A7 | CXL16_HUMAN |
| 1972 | Q9H2G2 | SLK_HUMAN |
| 1973 | Q9H2K0 | IF3M_HUMAN |
| 1974 | Q9H2R5 | KLK15_HUMAN |
| 1975 | Q9H2X3 | CLC4M_HUMAN |
| 1976 | Q9H3G5 | CPVL_HUMAN |
| 1977 | Q9H3K6 | BOLA2_HUMAN |
| 1978 | Q9H3R2 | MUC13_HUMAN |
| 1979 | Q9H3S3 | TMPS5_HUMAN |
| 1980 | Q9H3S4 | TPK1_HUMAN |
| 1981 | Q9H3T2 | SEM6C_HUMAN |
| 1982 | Q9H3U7 | SMOC2_HUMAN |
| 1983 | Q9H446 | RWDD1_HUMAN |
| 1984 | Q9H461 | FZD8_HUMAN |
| 1985 | Q9H477 | RBSK_HUMAN |
| 1986 | Q9H4A9 | DPEP2_HUMAN |
| 1987 | Q9H4D0 | CSTN2_HUMAN |
| 1988 | Q9H4F8 | SMOC1_HUMAN |
| 1989 | Q9H4P4 | RNF41_HUMAN |
| 1990 | Q9H4X1 | RGCC_HUMAN |
| 1991 | Q9H5V8 | CDCP1_HUMAN |
| 1992 | Q9H5Y7 | SLIK6_HUMAN |
| 1993 | Q9H6B4 | CLMP_HUMAN |
| 1994 | Q9H6E4 | CC134_HUMAN |
| 1995 | Q9H6Q3 | SLAP2_HUMAN |
| 1996 | Q9H6S1 | AZI2_HUMAN |
| 1997 | Q9H6S3 | ES8L2_HUMAN |
| 1998 | Q9H741 | SPRNG_HUMAN |
| 1999 | Q9H773 | DCTP1_HUMAN |
| 2000 | Q9H777 | RNZ1_HUMAN |
| 2001 | Q9H7C9 | AAMDC_HUMAN |
| 2002 | Q9H7M9 | VISTA_HUMAN |
| 2003 | Q9H7Y0 | DIK2B_HUMAN |
| 2004 | Q9H7Z7 | PGES2_HUMAN |
| 2005 | Q9H832 | UBE2Z_HUMAN |
| 2006 | Q9H867 | MT21D_HUMAN |
| 2007 | Q9H8J5 | MANS1_HUMAN |
| 2008 | Q9H910 | JUPI2_HUMAN |
| 2009 | Q9H939 | PPIP2_HUMAN |
| 2010 | Q9HA65 | TBC17_HUMAN |
| 2011 | Q9HAN9 | NMNA1_HUMAN |
| 2012 | Q9HAT2 | SIAE_HUMAN |
| 2013 | Q9HAV5 | TNR27_HUMAN |
| 2014 | Q9HAV7 | GRPE1_HUMAN |
| 2015 | Q9HB29 | ILRL2_HUMAN |
| 2016 | Q9HB40 | RISC_HUMAN |
| 2017 | Q9HB71 | CYBP_HUMAN |
| 2018 | Q9HBB8 | CDHR5_HUMAN |
| 2019 | Q9HBG7 | LY9_HUMAN |
| 2020 | Q9HC38 | GLOD4_HUMAN |
| 2021 | Q9HC56 | PCDH9_HUMAN |
| 2022 | Q9HC57 | WFDC1_HUMAN |
| 2023 | Q9HC77 | CENPJ_HUMAN |
| 2024 | Q9HCB6 | SPON1_HUMAN |
| 2025 | Q9HCK4 | ROBO2_HUMAN |
| 2026 | Q9HCM2 | PLXA4_HUMAN |
| 2027 | Q9HCN6 | GPVI_HUMAN |
| 2028 | Q9HCU0 | CD248_HUMAN |
| 2029 | Q9HCU4 | CELR2_HUMAN |
| 2030 | Q9HCY8 | S10AE_HUMAN |
| 2031 | Q9HD26 | GOPC_HUMAN |
| 2032 | Q9HD43 | PTPRH_HUMAN |
| 2033 | Q9HD89 | RETN_HUMAN |
| 2034 | Q9NNX6 | CD209_HUMAN |
| 2035 | Q9NP70 | AMBN_HUMAN |
| 2036 | Q9NP79 | VTA1_HUMAN |
| 2037 | Q9NP84 | TNR12_HUMAN |
| 2038 | Q9NPB3 | CABP2_HUMAN |
| 2039 | Q9NPG4 | PCD12_HUMAN |
| 2040 | Q9NPH0 | PPA6_HUMAN |
| 2041 | Q9NPH3 | IL1AP_HUMAN |
| 2042 | Q9NPH6 | OBP2B_HUMAN |
| 2043 | Q9NPJ3 | ACO13_HUMAN |
| 2044 | Q9NPY3 | C1QR1_HUMAN |
| 2045 | Q9NQ25 | SLAF7_HUMAN |
| 2046 | Q9NQ30 | ESM1_HUMAN |
| 2047 | Q9NQ38 | ISK5_HUMAN |
| 2048 | Q9NQ48 | LZTL1_HUMAN |
| 2049 | Q9NQ76 | MEPE_HUMAN |
| 2050 | Q9NQ79 | CRAC1_HUMAN |
| 2051 | Q9NQ84 | GPC5C_HUMAN |
| 2052 | Q9NQ88 | TIGAR_HUMAN |
| 2053 | Q9NQW8 | CNGB3_HUMAN |
| 2054 | Q9NQX5 | NPDC1_HUMAN |
| 2055 | Q9NR12 | PDLI7_HUMAN |
| 2056 | Q9NR28 | DBLOH_HUMAN |
| 2057 | Q9NR46 | SHLB2_HUMAN |
| 2058 | Q9NR71 | ASAH2_HUMAN |
| 2059 | Q9NRA1 | PDGFC_HUMAN |
| 2060 | Q9NRJ3 | CCL28_HUMAN |
| 2061 | Q9NRM6 | I17RB_HUMAN |
| 2062 | Q9NRR1 | CYTL1_HUMAN |
| 2063 | Q9NRS6 | SNX15_HUMAN |
| 2064 | Q9NRY6 | PLS3_HUMAN |
| 2065 | Q9NS15 | LTBP3_HUMAN |
| 2066 | Q9NS62 | THSD1_HUMAN |
| 2067 | Q9NS68 | TNR19_HUMAN |
| 2068 | Q9NS71 | GKN1_HUMAN |
| 2069 | Q9NS98 | SEM3G_HUMAN |
| 2070 | Q9NSA1 | FGF21_HUMAN |
| 2071 | Q9NSK7 | CS012_HUMAN |
| 2072 | Q9NTU7 | CBLN4_HUMAN |
| 2073 | Q9NUW8 | TYDP1_HUMAN |
| 2074 | Q9NUY8 | TBC23_HUMAN |
| 2075 | Q9NVZ3 | NECP2_HUMAN |
| 2076 | Q9NWM8 | FKB14_HUMAN |
| 2077 | Q9NWQ8 | PHAG1_HUMAN |
| 2078 | Q9NWZ3 | IRAK4_HUMAN |
| 2079 | Q9NXA8 | SIR5_HUMAN |
| 2080 | Q9NXH3 | PP14D_HUMAN |
| 2081 | Q9NXV2 | KCTD5_HUMAN |
| 2082 | Q9NY25 | CLC5A_HUMAN |
| 2083 | Q9NY59 | NSMA2_HUMAN |
| 2084 | Q9NYJ8 | TAB2_HUMAN |
| 2085 | Q9NYZ4 | SIGL8_HUMAN |
| 2086 | Q9NZ53 | PDXL2_HUMAN |
| 2087 | Q9NZA1 | CLIC5_HUMAN |
| 2088 | Q9NZC2 | TREM2_HUMAN |
| 2089 | Q9NZD4 | AHSP_HUMAN |
| 2090 | Q9NZK5 | ADA2_HUMAN |
| 2091 | Q9NZN3 | EHD3_HUMAN |
| 2092 | Q9NZN5 | ARHGC_HUMAN |
| 2093 | Q9NZP8 | C1RL_HUMAN |
| 2094 | Q9NZQ7 | PD1L1_HUMAN |
| 2095 | Q9NZS2 | KLRF1_HUMAN |
| 2096 | Q9NZT2 | OGFR_HUMAN |
| 2097 | Q9NZV1 | CRIM1_HUMAN |
| 2098 | Q9P000 | COMD9_HUMAN |
| 2099 | Q9P013 | CWC15_HUMAN |
| 2100 | Q9P0G3 | KLK14_HUMAN |
| 2101 | Q9P0K1 | ADA22_HUMAN |
| 2102 | Q9P0M4 | IL17C_HUMAN |
| 2103 | Q9P0V8 | SLAF8_HUMAN |
| 2104 | Q9P126 | CLC1B_HUMAN |
| 2105 | Q9P1Z2 | CACO1_HUMAN |
| 2106 | Q9P232 | CNTN3_HUMAN |
| 2107 | Q9P2J2 | TUTLA_HUMAN |
| 2108 | Q9P2M7 | CING_HUMAN |
| 2109 | Q9P2T1 | GMPR2_HUMAN |
| 2110 | Q9P2X3 | IMPCT_HUMAN |
| 2111 | Q9UBC9 | SPRR3_HUMAN |
| 2112 | Q9UBG3 | CRNN_HUMAN |
| 2113 | Q9UBM4 | OPT_HUMAN |
| 2114 | Q9UBP4 | DKK3_HUMAN |
| 2115 | Q9UBQ7 | GRHPR_HUMAN |
| 2116 | Q9UBR1 | BUP1_HUMAN |
| 2117 | Q9UBR2 | CATZ_HUMAN |
| 2118 | Q9UBT3 | DKK4_HUMAN |
| 2119 | Q9UBU3 | GHRL_HUMAN |
| 2120 | Q9UBV2 | SE1L1_HUMAN |
| 2121 | Q9UBW5 | BIN2_HUMAN |
| 2122 | Q9UBX1 | CATF_HUMAN |
| 2123 | Q9UBX7 | KLK11_HUMAN |
| 2124 | Q9UDT6 | CLIP2_HUMAN |
| 2125 | Q9UEW3 | MARCO_HUMAN |
| 2126 | Q9UFP1 | GAK1A_HUMAN |
| 2127 | Q9UGM5 | FETUB_HUMAN |
| 2128 | Q9UGN4 | CLM8_HUMAN |
| 2129 | Q9UGT4 | SUSD2_HUMAN |
| 2130 | Q9UH03 | SEPT3_HUMAN |
| 2131 | Q9UH65 | SWP70_HUMAN |
| 2132 | Q9UHC6 | CNTP2_HUMAN |
| 2133 | Q9UHD0 | IL19_HUMAN |
| 2134 | Q9UHD8 | SEPT9_HUMAN |
| 2135 | Q9UHF1 | EGFL7_HUMAN |
| 2136 | Q9UHF4 | I20RA_HUMAN |
| 2137 | Q9UHI8 | ATS1_HUMAN |
| 2138 | Q9UHL4 | DPP2_HUMAN |
| 2139 | Q9UHN6 | CEIP2_HUMAN |
| 2140 | Q9UHP3 | UBP25_HUMAN |
| 2141 | Q9UHX3 | AGRE2_HUMAN |
| 2142 | Q9UI42 | CBPA4_HUMAN |
| 2143 | Q9UIB8 | SLAF5_HUMAN |
| 2144 | Q9UII2 | ATIF1_HUMAN |
| 2145 | Q9UIK4 | DAPK2_HUMAN |
| 2146 | Q9UIM3 | FKBPL_HUMAN |
| 2147 | Q9UJ68 | MSRA_HUMAN |
| 2148 | Q9UJ70 | NAGK_HUMAN |
| 2149 | Q9UJ71 | CLC4K_HUMAN |
| 2150 | Q9UJ72 | ANX10_HUMAN |
| 2151 | Q9UJA9 | ENPP5_HUMAN |
| 2152 | Q9UJM8 | HAOX1_HUMAN |
| 2153 | Q9UJU6 | DBNL_HUMAN |
| 2154 | Q9UK05 | GDF2_HUMAN |
| 2155 | Q9UK23 | NAGPA_HUMAN |
| 2156 | Q9UK41 | VPS28_HUMAN |
| 2157 | Q9UK53 | ING1_HUMAN |
| 2158 | Q9UK85 | DKKL1_HUMAN |
| 2159 | Q9UKJ0 | PILRB_HUMAN |
| 2160 | Q9UKJ1 | PILRA_HUMAN |
| 2161 | Q9UKK9 | NUDT5_HUMAN |
| 2162 | Q9UKL0 | RCOR1_HUMAN |
| 2163 | Q9UKM9 | RALY_HUMAN |
| 2164 | Q9UKP3 | ITBP2_HUMAN |
| 2165 | Q9UKR3 | KLK13_HUMAN |
| 2166 | Q9UKU9 | ANGL2_HUMAN |
| 2167 | Q9UKW4 | VAV3_HUMAN |
| 2168 | Q9UKX5 | ITA11_HUMAN |
| 2169 | Q9UKY0 | PRND_HUMAN |
| 2170 | Q9UL46 | PSME2_HUMAN |
| 2171 | Q9ULA0 | DNPEP_HUMAN |
| 2172 | Q9ULD2 | MTUS1_HUMAN |
| 2173 | Q9ULI3 | HEG1_HUMAN |
| 2174 | Q9ULL4 | PLXB3_HUMAN |
| 2175 | Q9ULR5 | PAI2B_HUMAN |
| 2176 | Q9ULX7 | CAH14_HUMAN |
| 2177 | Q9UM07 | PADI4_HUMAN |
| 2178 | Q9UM47 | NOTC3_HUMAN |
| 2179 | Q9UMF0 | ICAM5_HUMAN |
| 2180 | Q9UMR7 | CLC4A_HUMAN |
| 2181 | Q9UMS0 | NFU1_HUMAN |
| 2182 | Q9UMX5 | NENF_HUMAN |
| 2183 | Q9UN19 | DAPP1_HUMAN |
| 2184 | Q9UNE0 | EDAR_HUMAN |

| | Gene | Protein | Gene | Protein | Gene | Protein | Gene | Protein |
|---|---|---|---|---|---|---|---|---|
| 2185 | Q9UNK0 | STX8_HUMAN | | | | | | |
| 2186 | Q9UNZ2 | NSF1C_HUMAN | | | | | | |
| 2187 | Q9UP79 | ATS8_HUMAN | | | | | | |
| 2188 | Q9UPW0 | FOXJ3_HUMAN | | | | | | |
| 2189 | Q9UQ16 | DYN3_HUMAN | | | | | | |
| 2190 | Q9UQB8 | BAIP2_HUMAN | | | | | | |
| 2191 | Q9UQP3 | TENN_HUMAN | | | | | | |
| 2192 | Q9UQQ2 | SH2B3_HUMAN | | | | | | |
| 2193 | Q9UQV4 | LAMP3_HUMAN | | | | | | |
| 2194 | Q9Y240 | CLC11_HUMAN | | | | | | |
| 2195 | Q9Y243 | AKT3_HUMAN | | | | | | |
| 2196 | Q9Y251 | HPSE_HUMAN | | | | | | |
| 2197 | Q9Y258 | CCL26_HUMAN | | | | | | |
| 2198 | Q9Y265 | RUVB1_HUMAN | | | | | | |
| 2199 | Q9Y266 | NUDC_HUMAN | | | | | | |
| 2200 | Q9Y275 | TN13B_HUMAN | | | | | | |
| 2201 | Q9Y279 | VSIG4_HUMAN | | | | | | |
| 2202 | Q9Y285 | SYFA_HUMAN | | | | | | |
| 2203 | Q9Y286 | SIGL7_HUMAN | | | | | | |
| 2204 | Q9Y2B0 | CNPY2_HUMAN | | | | | | |
| 2205 | Q9Y2E5 | MA2B2_HUMAN | | | | | | |
| 2206 | Q9Y2J8 | PADI2_HUMAN | | | | | | |
| 2207 | Q9Y2V2 | CHSP1_HUMAN | | | | | | |
| 2208 | Q9Y2W1 | TR150_HUMAN | | | | | | |
| 2209 | Q9Y2W6 | TDRKH_HUMAN | | | | | | |
| 2210 | Q9Y2X7 | GIT1_HUMAN | | | | | | |
| 2211 | Q9Y2Y0 | AR2BP_HUMAN | | | | | | |
| 2212 | Q9Y2Y8 | PRG3_HUMAN | | | | | | |
| 2213 | Q9Y2Z0 | SGT1_HUMAN | | | | | | |
| 2214 | Q9Y336 | SIGL9_HUMAN | | | | | | |
| 2215 | Q9Y3C0 | WASC3_HUMAN | | | | | | |
| 2216 | Q9Y3D6 | FIS1_HUMAN | | | | | | |
| 2217 | Q9Y3E2 | BOLA1_HUMAN | | | | | | |
| 2218 | Q9Y3L3 | 3BP1_HUMAN | | | | | | |
| 2219 | Q9Y3P8 | SIT1_HUMAN | | | | | | |
| 2220 | Q9Y478 | AAKB1_HUMAN | | | | | | |
| 2221 | Q9Y4D1 | DAAM1_HUMAN | | | | | | |
| 2222 | Q9Y4K4 | M4K5_HUMAN | | | | | | |
| 2223 | Q9Y4L1 | HYOU1_HUMAN | | | | | | |
| 2224 | Q9Y4X3 | CCL27_HUMAN | | | | | | |
| 2225 | Q9Y570 | PPME1_HUMAN | | | | | | |
| 2226 | Q9Y5A7 | NUB1_HUMAN | | | | | | |
| 2227 | Q9Y5C1 | ANGL3_HUMAN | | | | | | |
| 2228 | Q9Y5E8 | PCDBF_HUMAN | | | | | | |
| 2229 | Q9Y5K2 | KLK4_HUMAN | | | | | | |
| 2230 | Q9Y5K6 | CD2AP_HUMAN | | | | | | |
| 2231 | Q9Y5L3 | ENTP2_HUMAN | | | | | | |
| 2232 | Q9Y5Q6 | INSL5_HUMAN | | | | | | |
| 2233 | Q9Y5S2 | MRCKB_HUMAN | | | | | | |
| 2234 | Q9Y5W5 | WIF1_HUMAN | | | | | | |
| 2235 | Q9Y5X1 | SNX9_HUMAN | | | | | | |
| 2236 | Q9Y5Y7 | LYVE1_HUMAN | | | | | | |
| 2237 | Q9Y624 | JAM1_HUMAN | | | | | | |
| 2238 | Q9Y639 | NPTN_HUMAN | | | | | | |
| 2239 | Q9Y646 | CBPQ_HUMAN | | | | | | |
| 2240 | Q9Y653 | AGRG1_HUMAN | | | | | | |
| 2241 | Q9Y662 | HS3SB_HUMAN | | | | | | |
| 2242 | Q9Y680 | FKBP7_HUMAN | | | | | | |
| 2243 | Q9Y6A5 | TACC3_HUMAN | | | | | | |
| 2244 | Q9Y6D9 | MD1L1_HUMAN | | | | | | |
| 2245 | Q9Y6K9 | NEMO_HUMAN | | | | | | |
| 2246 | Q9Y6N7 | ROBO1_HUMAN | | | | | | |
| 2247 | Q9Y6Q6 | TNR11_HUMAN | | | | | | |
| 2248 | Q9Y6X8 | ZHX2_HUMAN | | | | | | |
| 2249 | Q9Y6Y9 | LY96_HUMAN | | | | | | |
| 2250 | NTproBNP | NPPB_HUMAN | | | | | | |

**Supplementary Table S3:** List and suppliers of the markers measured using immunoassay

| Protein | Supplier | Catalog Number |
|---|---|---|
| ACRP30 | Biotechne | DY1065 |
| CA125 | Merck Life Science AB | HCCBP1MAG-58K |
| CA153 | Merck Life Science AB | HCCBP1MAG-58K |
| CA199 | Merck Life Science AB | HCCBP1MAG-58K |
| CA50 | Creative Biolabs | CGYJ176, Creative BioMart CA50-01H |
| CEA | Merck Life Science AB | HCCBP1MAG-58K |
| CTSD | Proteomedix | CTSD IVD ELISA |
| DLK1 | Biotechne | DY1144 |
| ECM1 | Biotechne | DY3937 |
| GAL3BP | Biotechne | DY2226-05 |
| ICAM1 | Proteomedix | Propriatory Antibodies & Protein |
| IL-1Ra | Biotechne | DY280 |
| IL6 | Merck Life Science AB | HCCBP1MAG-58K |
| LAMC2 | Abcam | ab253606, STN-50429 |
| LCN2 | Biotechne | DY1757 |
| LRG1 | Biotechne | Custom made |
| LUM | Biotechne | DY2846 |
| MMP9 | Biotechne | DY911 |
| NCAM1 | Biotechne | DY2408 |
| OPG | Biotechne | DY805 |
| OPN | Merck Life Science AB | HCCBP1MAG-58K |
| POSTN | Proteomedix | Propriatory Antibodies & Protein |
| THBS1 | Proteomedix | THBS1 IVD ELISA |
| THBS2 | Biotechne | DY1635 |
| TIMP1 | Biotechne | DY970 |
| TTR | Abcam | ab244071, AB77905 |
| ZAG | Biotechne | DY4764 |

**Supplementary Table S4:** Clinical performance of the 25 marker combinations in the sub-populations.

| Combination | Marker 1 | Marker 2 | Marker 3 | Marker 4 | Marker 5 | Marker 6 | Target Pop. Sen. (%) at 95% Spe. | Low CA19-9 Pop. Sen. (%) at 95% Spe. | CP Pop. Sen. (%) at 96% Spe. | NOD Pop. Sen. (%) at 96% Spe. |
|---|---|---|---|---|---|---|---|---|---|---|
| Reference | CA19-9 | - | - | - | - | - | 53 | 0 | 31 | 41 |
| combination 1 | - | MXRA8 | LTBP2 | PLA2G1B | KIRREL2 | - | 84 | 77 | 4 | 0 |
| combination 2 | - | MXRA8 | LTBP2 | PLA2G1B | IL7R | - | 81 | 74 | 4 | 0 |
| combination 3 | CA19-9 | MXRA8 | MMP7 | PPY | - | - | 80 | 58 | 3 | 18 |
| combination 4 | - | MXRA8 | LTBP2 | PLA2G1B | SEMA7A | - | 79 | 68 | 4 | 0 |
| combination 5 | CA19-9 | MXRA8 | MMP7 | PLA2G1B | - | - | 76 | 61 | 36 | 9 |
| combination 6 | - | KLK10 | CPB1 | PLA2G1B | CTRL | - | 75 | 77 | 41 | 50 |
| combination 7 | - | MXRA8 | LTBP2 | PLA2G1B | ADAMTS16 | - | 75 | 68 | 5 | 0 |
| combination 8 | CA19-9 | KLK10 | CPB1 | PLA2G1B | | - | 73 | 71 | 31 | 56 |
| combination 9 | CA19-9 | MUC13 | CLPS | GPA33 | - | - | 73 | 39 | 27 | 46 |
| combination 10 | CA19-9 | REG4 | TTR | KIRREL2 | - | - | 72 | 48 | 1 | 32 |
| combination 11 | CA19-9 | TIMP1 | CA153 | CTSD | - | - | 71 | 71 | 9 | 64 |
| combination 12 | CA19-9 | AHNAK | MUC13 | PLA2G1B | MERTK | TIMP2 | 68 | 39 | 49 | 55 |
| combination 13 | CA19-9 | TIMP1 | CA153 | CEA | - | - | 68 | 71 | 13 | 32 |
| combination 14 | - | MUC13 | GPNMB | CEA | PI16 | - | 68 | 48 | 8 | 9 |
| combination 15 | CA19-9 | KLKB1 | ANGTPL2 | AHNAK | - | - | 67 | 36 | 16 | 73 |
| combination 16 | CA19-9 | TIMP2 | CPB1 | PLA2G1B | - | - | 67 | 45 | 7 | 23 |
| combination 17 | - | SDF1 | CPB1 | PLA2G1B | MERTK | - | 64 | 61 | 8 | 50 |
| combination 18 | CA19-9 | ICAM1 | TTR | OPN | - | - | 64 | 58 | 8 | 5 |
| combination 19 | CA19-9 | MUC13 | TTR | PTPRK | - | - | 63 | 32 | 3 | 32 |
| combination 20 | CA19-9 | NEFL | SUOX | PLA2G1B | - | - | 60 | 26 | 31 | 36 |
| combination 21 | CA19-9 | ICAM1 | TIMP1 | THBS1 | CTSD | - | 60 | 52 | 15 | 18 |
| combination 22 | CA19-9 | ICAM1 | TIMP1 | THBS1 | - | - | 60 | 52 | 9 | 18 |
| combination 23 | CA19-9 | ICAM1 | TIMP1 | CTSD | - | - | 59 | 45 | 9 | 18 |
| combination 24 | - | LIPR1 | VEGFC | PLA2G1B | TIMP1 | - | 52 | 84 | 3 | 5 |
| combination 25 | CA19-9 | FLT3L | FGL1 | TR10A | - | - | 51 | 84 | 0 | 5 |
